# Spatially resolved single-cell atlas unveils a distinct cellular signature of fatal lung COVID-19 in a Malawian population

**DOI:** 10.1038/s41591-024-03354-3

**Published:** 2024-11-20

**Authors:** James Nyirenda, Olympia M. Hardy, João Da Silva Filho, Vanessa Herder, Charalampos Attipa, Charles Ndovi, Memory Siwombo, Takondwa Rex Namalima, Leticia Suwedi, Georgios Ilia, Watipenge Nyasulu, Thokozile Ngulube, Deborah Nyirenda, Leonard Mvaya, Joseph Phiri, Dennis Chasweka, Chisomo Eneya, Chikondi Makwinja, Chisomo Phiri, Frank Ziwoya, Abel Tembo, Kingsley Makwangwala, Stanley Khoswe, Peter Banda, Ben Morton, Orla Hilton, Sarah Lawrence, Monique Freire dos Reis, Gisely Cardoso Melo, Marcus Vinicius Guimaraes de Lacerda, Fabio Trindade Maranhão Costa, Wuelton Marcelo Monteiro, Luiz Carlos de Lima Ferreira, Carla Johnson, Dagmara McGuinness, Kondwani Jambo, Michael Haley, Benjamin Kumwenda, Massimo Palmarini, Donna M. Denno, Wieger Voskuijl, Steve Bvuobvuo Kamiza, Kayla G. Barnes, Kevin Couper, Matthias Marti, Thomas D. Otto, Christopher A. Moxon

**Affiliations:** 1 School of Infection and Immunity, College of Medical Veterinary and Life Sciences, University of Glasgow, Glasgow, UK; 2 Blantyre Malaria Project, Kamuzu University of Health Sciences, Blantyre, Malawi; 3 Malawi-Liverpool-Wellcome Programme, Kamuzu University of Health Sciences, Blantyre, Malawi; 4 Universität Zürich, Institut für Parasitologie, Zurich, Switzerland; 5 MRC-University of Glasgow Centre for Virus Research, Glasgow, UK; 6 The Royal (Dick) School of Veterinary Studies, University of Edinburgh, Edinburgh, UK; 7 The Roslin Institute, University of Edinburgh, Edinburgh, UK; 8 Queen Elizabeth Central Hospital, Blantyre, Malawi; 9 Kamuzu University of Science of Health Sciences, Blantyre, Malawi; 10 International Public Health, Liverpool School of Tropical Medicine, Liverpool, UK; 11 Department of Clinical Sciences, Liverpool School of Tropical Medicine, Liverpool, UK; 12 Department of Infectious Diseases, Imperial College London, London, UK; 13 Department of Global Health and Pediatrics, University of Washington, Seattle, WA USA; 14 Department of Education and Research, Oncology Control Centre of Amazonas State (FCECON), Manaus, Brazil; 15 Postgraduate Program in Tropical Medicine, University of Amazonas State, Manaus, Brazil; 16 Tropical Medicine Foundation Dr. Heitor Vieira Dourado, Manaus, Brazil; 17 Institute Leônidas & Maria Deane, Fiocruz, Manaus, Brazil; 18 The University of Texas Medical Branch, Galveston, TX USA; 19 Department of Genetics, Evolution, Microbiology and Immunology, University of Campinas, Campinas, Brazil; 20 Division of Immunology, Immunity to Infection & Respiratory Medicine, Faculty of Biology, University of Manchester, Manchester, UK; 21 Department of Global Health, Amsterdam University Medical Centers, Amsterdam, The Netherlands; 22 Department of Tropical Disease Biology, Liverpool School of Tropical Medicine, Liverpool, UK; 23 Department of Immunology and Infectious Diseases, Harvard T.H. Chan School of Public Health, Boston, MA USA; 24 Broad Institute of MIT and Harvard, Cambridge, MA USA; 25 Department of Vector Biology, Liverpool School of Tropical Medicine, Liverpool, UK

**Keywords:** Infection, Immunopathogenesis, Viral infection, Single-cell imaging

## Abstract

Postmortem single-cell studies have transformed understanding of lower respiratory tract diseases (LRTDs), including coronavirus disease 2019 (COVID-19), but there are minimal data from African settings where HIV, malaria and other environmental exposures may affect disease pathobiology and treatment targets. In this study, we used histology and high-dimensional imaging to characterize fatal lung disease in Malawian adults with (*n* = 9) and without (*n* = 7) COVID-19, and we generated single-cell transcriptomics data from lung, blood and nasal cells. Data integration with other cohorts showed a conserved COVID-19 histopathological signature, driven by contrasting immune and inflammatory mechanisms: in US, European and Asian cohorts, by type I/III interferon (IFN) responses, particularly in blood-derived monocytes, and in the Malawian cohort, by response to IFN-γ in lung-resident macrophages. HIV status had minimal impact on histology or immunopathology. Our study provides a data resource and highlights the importance of studying the cellular mechanisms of disease in underrepresented populations, indicating shared and distinct targets for treatment.

## Abstract (web summary)

A single-cell study integrating data from lung tissues from patients with fatal COVID-19 from Malawi, the United States and Europe identifies shared and distinct immune and inflammatory mechanisms of response.

## Main

Progress toward a human cell atlas (HCA) using single-cell RNA-sequencing (scRNA-seq) and high-dimensional imaging is transforming understanding of cells and their states in health and disease and is rapidly becoming a major resource for the development of novel treatments and vaccines^1–3^. However, data within this atlas are heavily biased toward populations in the Northern Hemisphere. Populations in sub-Saharan Africa (SSA) are particularly underrepresented^4^. Genetic and environmental factors may lead to important differences in cell development and cell compositions in different organs, thus affecting cellular responses to diseases, vaccines and therapies^5,6^. Capturing data from SSA populations is critical to assure more equitable benefit from the treatment advances derived from the HCA.

Immunomodulation plays a critical role in coronavirus disease 2019 (COVID-19) outcomes. Single-cell data from lung tissue facilitated identification of specific immunomodulatory targets^6–12^. Apart from our high-dimensional imaging data from a Brazilian cohort^13^, single-cell data are restricted to populations in the Northern Hemisphere, such as clinical trial data validating their efficacy^14–16^. For future outbreaks of severe acute respiratory syndrome coronavirus 2 (SARS-CoV-2) or related viruses, this knowledge gap needs to be addressed. Indeed, given fewer intensive care facilities, the benefit of immunomodulation for severe disease is even more important in SSA. Although immunomodulatory therapies can be lifesaving, they can also be harmful^16^. Immunomodulation has broadly focused on two opposing strategies: augmenting inflammatory responses to aid viral clearance or attenuating inflammatory response to reduce hyperinflammation. Extensive studies in Northern Hemisphere cohorts have established that, by the time patients present with life-threatening illness, viral loads are declining, hyperinflammation generally predominates and, thus, anti-inflammatory interventions are more effective^15,16^. Given evidence that repeated exposure to malaria and other parasitic infections can induce immune tolerance^17,18^, and because parasitic infections occur at higher levels in SSA populations^19–22^, we hypothesized that the immune balance may be different in patients in SSA. Although sometimes this clinical context may be protective, in those who progress to severe disease a tolerance-skewed response might blunt immune-mediated viral clearance, leading to a more viral-driven pathology. However, the reverse is also possible. High pathogen exposure can induce an accelerated inflammatory response on re-exposure to pathogens^5^. Either scenario might impact cellular responses in the lung and have important implications to inform treatment choices in SSA populations. To address some of these knowledge gaps, we conducted an autopsy study in well-characterized patients at a large public hospital in Malawi, a low-income country in SSA with high rates of malaria, tuberculosis (TB) and HIV.

## Results

### A conserved histological signature of COVID-19 in Malawian patients

**Fig. 1:**
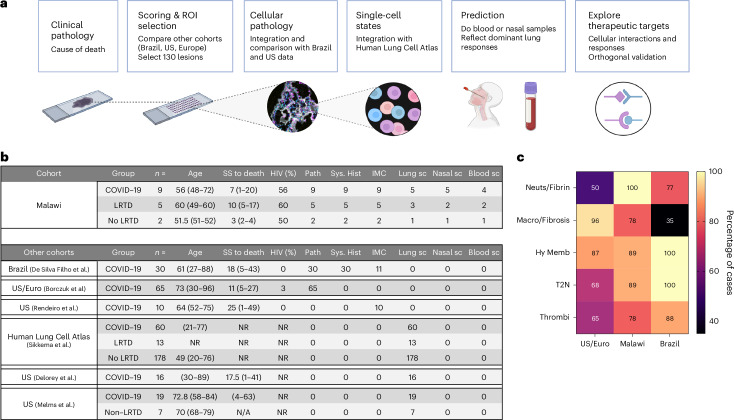
Study overview, overview of our cohort and comparator cohorts and histological lesion comparison with other cohorts. **a**, Overview of study approach, created with BioRender.com. **b**, Summary of the characteristics of our Malawian cohort versus published cohorts that we have used for different comparisons. **c**, Heatmap shows the proportion of patients in the three cohorts (US/European, Malawian and Brazilian) who have each given lesion type. SS to death, symptom start to death in days; Path, pathology, denotes the number of patients included in each cohort in which postmortem pathological features are described; Sys. Hist., systematic histopathology, denotes the number of patients included in each cohort with scoring of the frequency and severity of different lesions scored based on pre-defined criteria; IMC, imaging mass cytometry, denotes the number of patients with data for this; Lung sc, lung cell single-cell RNA-seq, denotes the number of patients with scRNA-seq data from lung tissue; Nasal sc, nasal cell single-cell RNA-seq, denotes the number of patients with scRNA-seq data from nasal tissue; Blood sc, blood cell single-cell RNA-seq, denotes the number of patients with these data. Hy Memb, hyline membranes; Macro, macrophages; N/A, not applicable; NR, not recorded; Neuts, neutrophilis; T2N, type II pneumocyte hyperplasia.

We recruited patients with fatal illness aged 45–75 years who were admitted to Queen Elizabeth Central Hospital (QECH), Blantyre, from October 2020 to July 2021 and stratified them into three groups based on clinical criteria: (1) COVID-19 acute respiratory distress syndrome (ARDS) (*n* = 9); (2) lower respiratory tract disease (LRTD) (*n* = 5) with ARDS of diverse non-COVID-19 etiology; and (3) non-LRTD (*n* = 2) (Fig. 1a,b, Extended Data Table 1, Supplementary Table 1 and Methods). Most patients with COVID-19 were overweight or obese (78%), and four had type 2 diabetes (44%). Patients with LRTD and non-LRTD patients were generally underweight. HIV infection was common across groups: five patients with COVID-19 (56%), three patients with LRTD (60%) and two non-LRTD patients (67%) had been living with HIV. In three patients, this diagnosis was not known during life; the other six had been on antiretroviral treatment, although drug availability was limited during the pandemic. All patients had low CD4 counts (median, 134 cells per mm^3^) (Extended Data Table 1 and Supplementary Tables 1 and 2).

**Extended Data Fig. 1:**
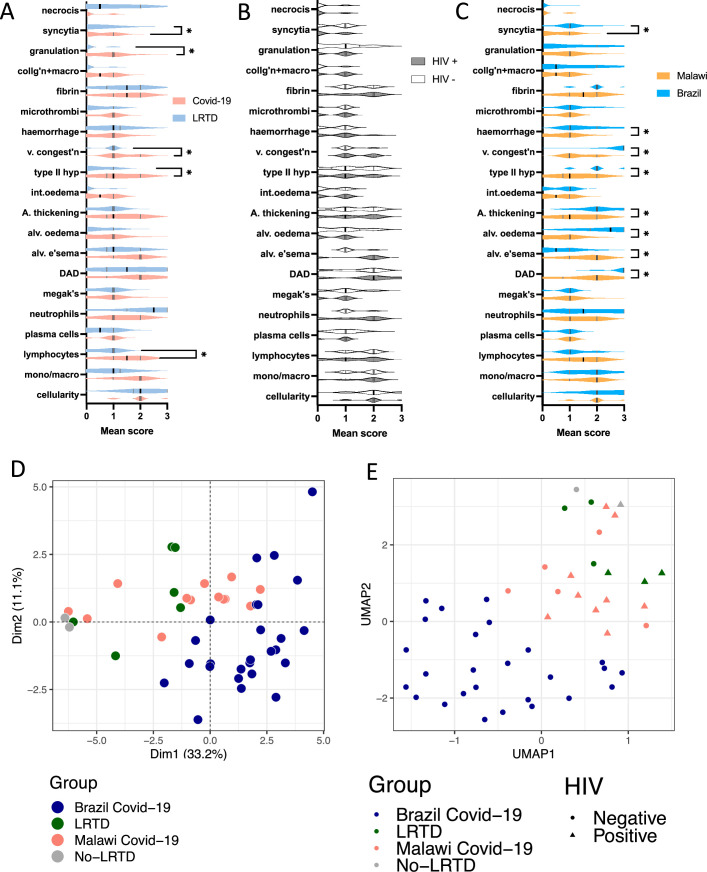
The histopathology of fatal Covid19 versus fatal non-Covid19 LRTD and non-LRTD in Malawian cases. Histopathology in the left and right lungs of the 16 cases was scored systematically using pre-defined criteria by two pathologists who were blinded to clinical information. We used identical scoring to a Brazil cohort that we have published on separately. A – C are violin plots of the distribution of scores to highlight comparisons between different group, central thick bars highlight the median and outer bars the interquartile range. In all three graphs a two-sided unpaired t-test was used to compare lesion frequencies with no correction for multiple comparisons * denotes p < 0.05 with specific p values given below. (**a**) Comparison of histological features between COVID-19 (n = 9) and non-COVID-19 fatal lower respiratory tract disease (LRTD) cases (n = 5). p values for significant individual comparisons: syncytia p = 0.008; type II hyperplasia p = 0.016; vascular congestion p = 0.031; lymphocytes p = 0.0032; granulation p = 0.034. (**b**) Comparison of histological features between HIV + COVID-19 cases (n = 5) and HIV- COVID-19 cases (n = 4). No comparisons had a p < 0.05 (**c**) Comparison of COVID-19 cases from Malawi cohort (n = 9) with cases from Brazil cohort (n = 20). p values for significant individual comparisons; vascular congestion, alveolar oedema DAD p = <0.00001; Alveolar thickening p = 0.0004; haemorrhage p = 0.0008; type II hyperplasia p = 0.0013; alveolar emphysema p = 0.0015; syncytia p = 0.0039. (**d**) PCA of cases split by groups. (**e)** UMAP of same data, including HIV status.

Using minimally invasive autopsy^23–25^, we obtained lung, liver and brain samples in 16 patients, bone marrow in 15 patients and spleen in eight patients. A pathologist read hematoxylin and eosin (H&E)-stained tissue slides alongside patients’ history and antemortem laboratory results. In the lung, the pathologist identified classical features of COVID-19 (refs. ^26–32^), which were less frequent in patients with LRTD (Supplementary Fig. 1 and Supplementary Table 3). COVID-19-specific changes were absent in other organs, focusing our further investigations on the lung. Then, two additional pathologists, blinded to diagnosis, scored the lung pathology in all 16 patients using more detailed semi-quantitative criteria^13^. In our patients with COVID-19, type II pneumocyte hyperplasia, vascular congestion, syncytia, granulation of tissue and lymphocyte infiltration were more common and severe than in the non-COVID-19 LRTD group (Extended Data Fig. 1a). No significant histopathology differences were observed due to HIV status (Extended Data Fig. 1b).

Lack of international consensus in COVID-19 lung pathology criteria, and of studies with systematic scoring, prevented quantitative comparison with other cohorts to assess similarities and differences. Therefore, we compared proportions of pulmonary lesion types with a study that combined cohorts from Europe and the United States (US)^27^ and with our published Brazilian cohort^13^ (Fig. 1c and Extended Data Fig. 1c). Acute alveolar changes, defined by neutrophil infiltration and fibrin deposition, were more frequent in the Malawian and Brazilian cohorts than in the US cohort. ‘Chronic’ alveolar changes with monocytes, macrophages or fibrosis were detected more frequently in the US and Malawian cohorts. In the Malawian cohort, ‘chronic’ disease was predominantly characterized by macrophage and monocytes with less fibrosis than in the US and Brazilian cohorts. Thus, despite a short duration from illness to death and demographic differences, patients in our Malawian cohort exhibited classical COVID-19 lung pathology but with a macrophage predominance in alveolar lesions.

### Resident macrophages predominate in COVID-19 and neutrophils in LRTD

**Fig. 2:**
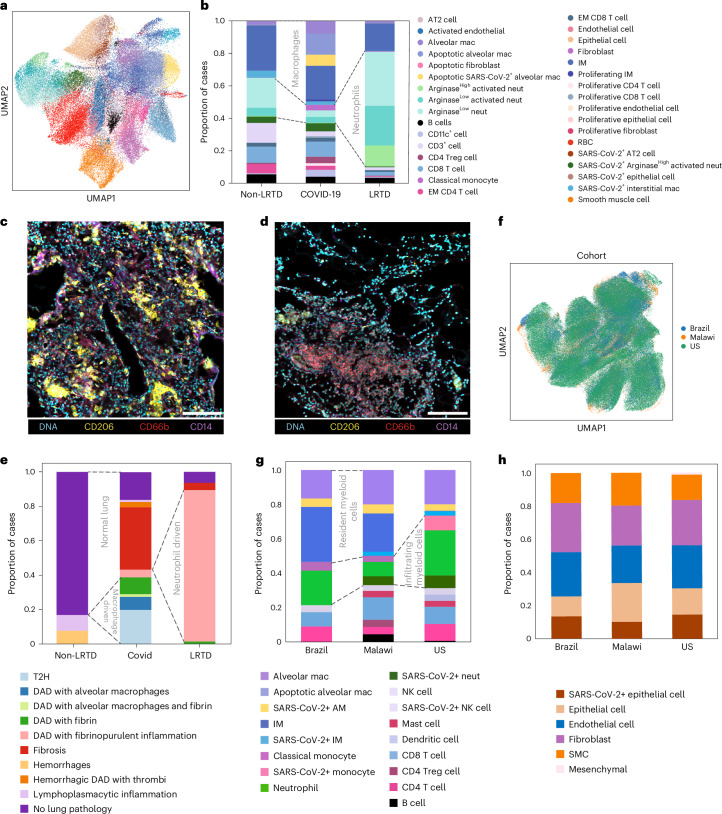
IMC reveals an immunopathological landscape of COVID-19 in Malawian patients driven by alveolar macrophages. **a**, UMAP embedding of the cell types identified in the lung samples by IMC, after supervised assignment to major cell types. Each major cell type was clustered, and resulting clusters were annotated and merged to extract the final set of cell types. Color key for cell types is on the right-hand side of **b**. Frequency of the immune cell types was identified in the postmortem lung samples by IMC according to clinical groups. The stacked bar plot shows the averaged frequency of the cell types by grouping the values from ROIs according to the clinical groups. Dashed lines highlight principal differences in major cell populations between COVID-19 and other respiratory disease groups. **c**, Representative denoised IMC images from one of 84 ROIs for patients with COVID-19 show abundant CD206^high^ macrophages (yellow) and few neutrophils (CD66b, red) and monocytes (CD14, purple). Scale bar, 140 μm. **d**, Representative denoised IMC images from one of 19 ROIs for a non-COVID-19 LRTD case show abundant neutrophils (CD66b, red) and few CD206^+^ macrophages (yellow). Scale bar, 140 μm. **e**, Frequency of histopathological lesions based on matched H&E and IMC analysis of postmortem lung samples from the different clinical groups. The cellular composition and frequency of different cell types are indicated in Extended Data Fig. 3f. Dotted lines highlight the differences in proportions of broad response categories. **f**, UMAP embedding shows good integration (using the scvi-tools package) of IMC lung datasets from the Brazilian, US and Malawian COVID-19 cohorts based on 17 common antibody markers. **g**, Comparison of immune cell frequencies in IMC data from Brazilian, Malawian and US cohorts after integration shown in **f**; some major cell group differences are highlighted by dotted lines. Dashed box highlights apoptotic alveolar macrophages that are present only in the Malawian cohort. **h**, Comparison of stromal cell frequencies in IMC data from Brazilian, Malawian and US cohorts after integration shown in **f**. AM, alveolar macrophage; DAD, diffuse alveolar macrophage; EM, effector memory; IM, interstitial macrophage, mac, macrophage; neut, neutrophil; NK, natural killer; Treg, regulatory T; T2N, type II pneumocyte hyperplasia.

**Extended Data Fig. 2:**
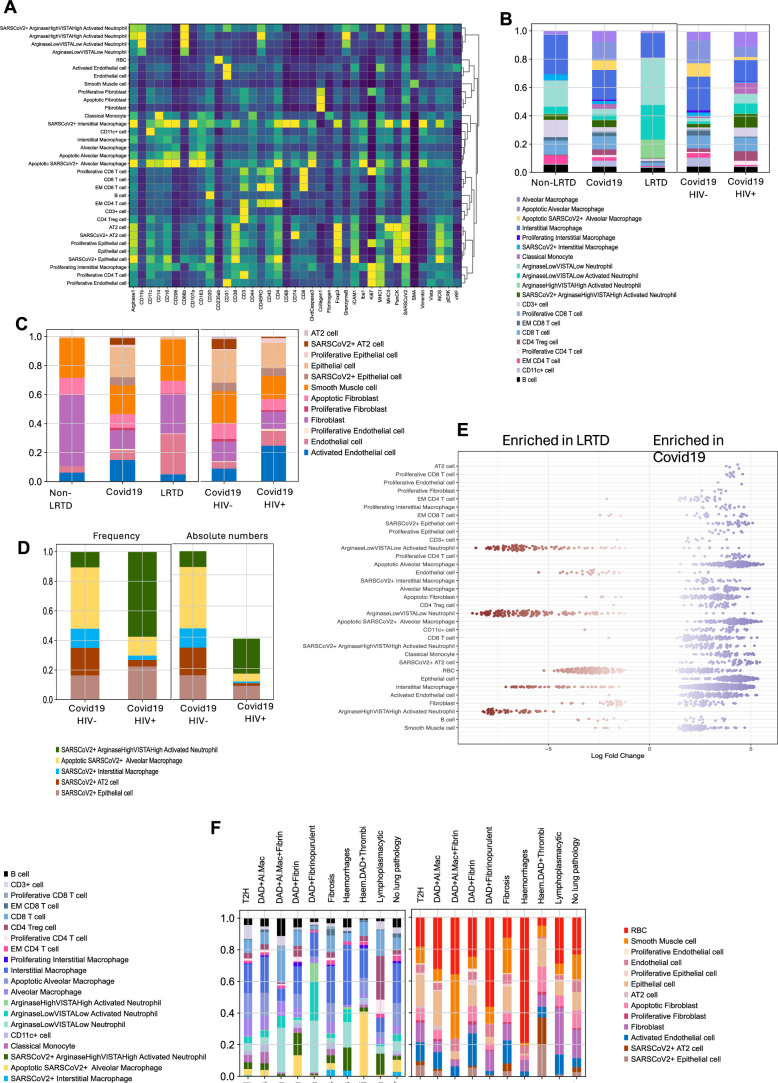
Cell atlas and phenotype of cell types identified in the post-mortem lung tissue determined by Imaging Mass Cytometry (IMC). **a**) Phenotype representation of each cell type identified in the lung samples. The heatmap shows the mean expression of each protein marker in the IMC panel in each cell type identified in the post-mortem lung tissue. (**b**) Frequency of the immune cell types identified in the post-mortem lung samples by IMC according to clinical groups and according to HIV status within the COVID-19 group. (**c**) Frequency of the stromal cell types identified in the post-mortem lung samples by IMC according to clinical groups and according to HIV status within the COVID-19 group. (**d**) Frequency and absolute numbers of SARS-CoV-2 Ag+ cells in the myeloid and epithelial compartments, determined by IMC, in the post-mortem lung samples according to HIV status within the COVID-19 group. (**e**) Cell type enrichment analysis of the cell populations identified in Malawi lung IMC data. The comparison shown is between COVID-19 versus LRTD cases. To correct for multiple testing, the spatial false discovery rate (FDR) was calculated and only dots with spatial FDR < 0.05 are shown. (**f**) Cellular landscape of histopathological lesions based on matched H&E and IMC analysis of post-mortem lung samples from the different clinical groups. The lesions were pooled, and the graph shows the average proportion of each cell type in each lesion type.

To assess pathology at the cellular level, tissue microarrays (TMAs) from 130 representative regions of interest (ROIs) from nine patients with COVID-19, three patients with LRTD and two non-LRTD patients, containing specific pathological lesions or normal lung areas, were analyzed by imaging mass cytometry (IMC). We used a 39-antibody panel optimized for staining in lung tissue^13^. After cell segmentation and quality control, 76,369 cells were annotated from 118 ROIs and classified into subtypes (Fig. 2a, Extended Data Fig. 2a–d and Supplementary Table 4).

In our Malawian cohort, neutrophils (CD66b^pos^CD11b^pos^CD14^neg/low^) were significantly more numerous in the patients with LRTD (49.6%) than in non-LRTD patients (21.1%) or in patients with COVID-19 (16.1%, *P* ≤ 0.001) (Fig. 2b,d, Extended Data Fig. 2e and Supplementary Table 4). Reciprocally, macrophages were increased in patients with COVID-19 (44.1%) compared to patients with LRTD (30.4%) and non-LRTD patients (23.6%; *P* ≤ 0.0001, Fig. 2b,c, Extended Data Fig. 2e and Supplementary Table 4). In contrast to data in prior published US and European cohorts^32,33^, these were predominantly tissue-resident alveolar macrophages (CD206^high^CD163^high^Iba1^low^MHCII^low^CD14^neg^) with a lower proportion of monocyte-derived CD14^high/int^ macrophages.

No consistent differences were observed in T cell numbers among the COVID-19, LRTD and non-LRTD disease groups, but, among patients with COVID-19, there was an expansion in regulatory T cells and proliferating T cells and a decrease in the ratio of effector memory (CD45RO^high^) to naive (CD45RO^low^) CD8 T cells (Fig. 2b and Supplementary Table 4). B cell numbers were not markedly different in COVID-19, although our panel had few markers to characterize B cells (Fig. 2b). Consistent with vascular pathology visualized by histology (fibrin deposition and thrombosis), there was increased endothelial cell activation in patients with COVID-19 compared to patients with LRTD and non-LRTD patients (Fig. 2b). Alveolar macrophages were the most common SARS-CoV-2^+^ immune cell, followed by Arg^high^ neutrophils and interstitial macrophages (Fig. 2b and Supplementary Table 4). In the stromal compartment, type II pneumocytes (AT2) and epithelial cells were the most frequent SARS-CoV-2^+^ cells. We found no SARS-CoV-2^+^ endothelial cells or fibroblasts. Surprisingly, total numbers of SARS-CoV-2^+^ cells were lower in HIV^+^ patients (Extended Data Fig. 2d and Supplementary Table 4).

Exploiting the spatial and cellular resolution of IMC, we characterized cellular compositions of lesion types (Extended Data Fig. 2f) and then quantified lesion type levels by group (Fig. 2e). Type II pneumocyte hyperplasia was specific to the COVID-19 group. Diffuse alveolar damage occurred in both LRTD and COVID-19 but had different compositions, indicating different pathological processes: in LRTD, with neutrophil-driven fibrinopurulent inflammation; in COVID-19, a more heterogeneous immune cell composition, dominated by the presence of macrophages, except fibrin-containing lesions, which were neutrophilic. Together, these data implicate macrophages in alveolar damage and neutrophils in vascular damage and coagulopathic processes.

### Common and unique myeloid compositions across cohorts

To systematically compare data across cohorts, we integrated IMC data from the Malawian patients with COVID-19 (*n* = 9) with our Brazilian cohort (*n* = 11) that employed the same antibody panel^13^ and a US cohort^34^ (*n* = 10) that used several of the same markers (Fig. 2f). Many similarities in cell proportions were observed among the three cohorts but also important differences (Fig. 2g,h and Supplementary Table 4). In the myeloid compartment, the Malawian and Brazilian cohorts were dominated by high levels of alveolar and interstitial macrophages compared to the US patients (Malawi 44.1%; Brazil 51.9%; US 26.3%). Reciprocally, the US cohort had the highest proportion of neutrophils (34.1%), and the Malawian cohort had the fewest (16.7%; Brazil 21.4%; *P* < 4.25 × 10^−9^). The proportion of B cells was also significantly higher in the Malawian cohort (6.89%; Brazilian cohort <0.1%; US cohort 0.7%; *P* = 1.85 × 10^−16^). In the stromal compartments, there was a lower proportion of fibroblasts in the Malawian cohort, in keeping with lower levels of fibrosis on histology.

SARS-CoV-2 antigen gives an indication of the quantity of viral material, although it does not distinguish replicating virus. The US cohort had the highest number of SARS-CoV-2^+^ immune cells (23.7%; Malawi 7.6%; Brazil 8.3%). These were principally monocytes and neutrophils in the US cohort versus CD206^high^ tissue-resident alveolar macrophages and neutrophils in the Malawian cohort and interstitial macrophages in the Brazilian cohort (Fig. 2g and Supplementary Table 4). In the stromal compartment, SARS-CoV-2 was detected in epithelial cells in all three cohorts but was significantly lower in the Malawian cohort (Fig. 2h; 5.8%, *P* = 1.18 × 10^−10^) compared to the Brazilian (13.1%) and US (12.1%) cohorts.

**Extended Data Fig. 3:**
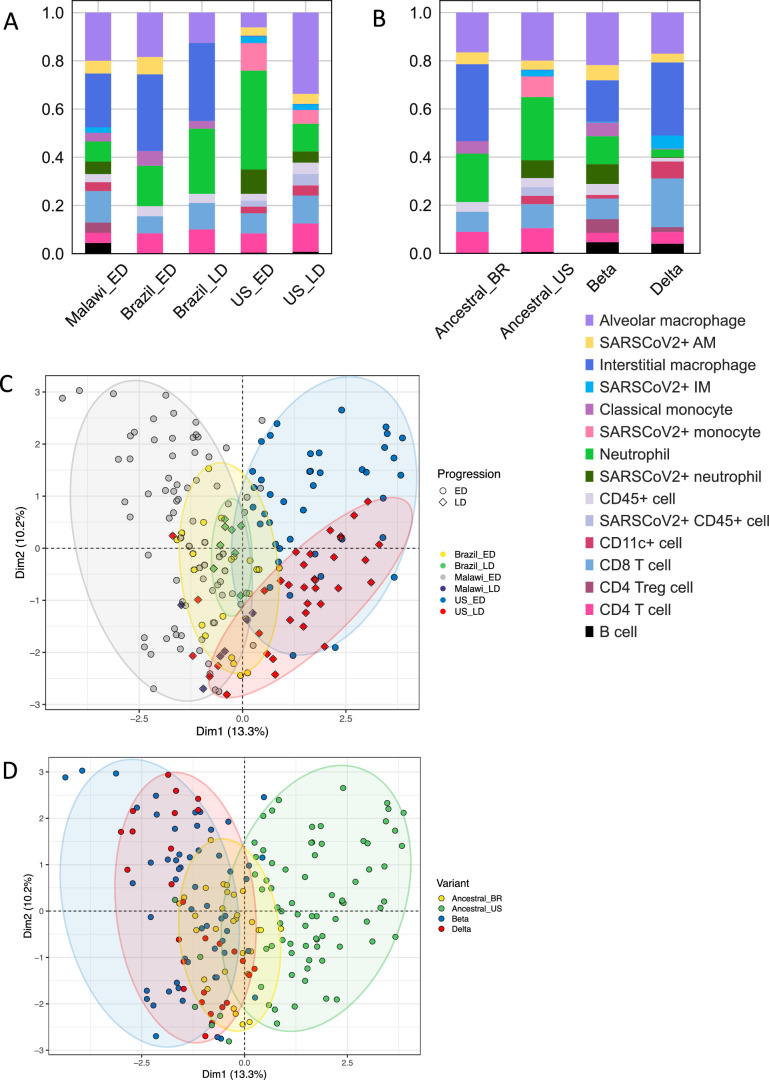
Effect of disease stage and viral variant on lung immune composition. **(a**,**b)** Immune cell proportions from lung imaging mass cytometry data in: **(a)** the Malawi cohort versus Brazil and USA early death (ED; that is cases who died within two weeks of illness onset) and late death (LD; that is cases who died after 2 weeks of illness). When compared with Malawi cases, USA early death (ED) have an even higher proportion of neutrophils and a lower proportion of macrophages than late death (LD). **(b)** Lung proportions in Brazil and USA cohorts who had the ancestral variant compared with Malawi cases with Beta and Malawi cases with Delta variant. The low proportion of neutrophils and high proportion of CD206^high^ macrophages in the Malawi cases are present regardless of variant. (**c, d**) Principal component analysis of of IMC lung immune cell composition data where cases are coloured by geographical location of the cohort (Malawi, Brazil, USA) and either: (**c**) disease stage (early versus late death) or (**d**) viral variant. Each dot is a separate lung sample from a different lung region from tissue microarrays (Malawi and USA) or lung blocks (Brazil). Coloured oval areas indicate where the majority of samples from each group cluster drawn by visual estimation using the same colours as those for the dots as indicated in the legend. For both graphs samples cluster principally by geographical location of the cohort and not by disease stage or viral variant.

A possible explanation for different myeloid compositions in Malawian versus Brazilian and US patients is illness duration. Previous COVID-19 studies demonstrated that patients dying within 2 weeks of illness onset (early death) have different immune responses from those dying after 2 weeks (late death)^13,33^. Only one Malawian case was late death, and the median illness duration before death was shorter than in US and Brazilian cohorts (Fig. 1b). Therefore, instead, we compared early versus late death US patients. If illness duration was a major driver of cell compositions, myeloid cell proportions in early death US patients should be more like the Malawian patients (lower neutrophils and monocytes, higher macrophages). Instead, early death US patients had an even higher proportion of neutrophils (40.4% US early, 13.7% US late, 13.4% Malawi) and monocytes (11.1% US early, 7.0% US late, 3.8% Malawi) and a lower proportion of lung-resident macrophages (15.0% early versus 40.1% late, versus 44.1% Malawi early) (Extended Data Fig. 3a and Supplementary Table 4). Furthermore, on a dimension reduction plot, samples clustered by population (Malawian, US and Brazilian) rather than by illness duration (early versus late) (Extended Data Fig. 3c). SARS-CoV-2 variant may also be an important driver of variance, as all US and Brazilian patients were of the ancestral variant, whereas Malawian patients were a mixture of Beta and Delta variant. However, cell proportions between patients with Beta or Delta variants in the Malawian patients were similar (Extended Data Fig. 3b). Furthermore, on a dimension reduction plot, we observed grouping by population, not viral variant (Extended Data Fig. 3d), suggesting that population is the main driver of lung immune composition.

Contrary to our initial hypothesis of a tolerized immune response in SSA populations, we found a highly inflammatory response and low levels of viral antigen in the Malawian patients versus other cohorts. The prominence of alveolar macrophages in lung lesions and enrichment of CD206^high^ tissue-resident macrophages in Malawian patients prompted further investigation of the inflammatory response in these cells.

### scRNA-seq reveals an interferon-gamma-dominated lung macrophage response

**Fig. 3:**
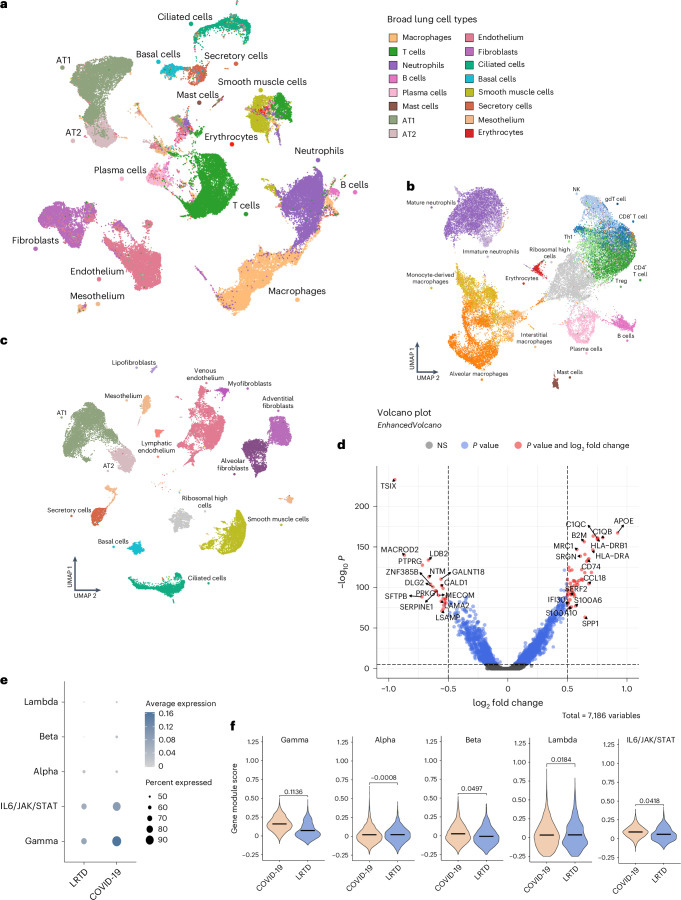
Lung single-cell atlas highlights IFN-γ response in alveolar macrophages. **a**, UMAP visualization of 66,882 lung cells across our cohort, colored by broad cell types cluster. **b**, UMAP visualization of 29,217 lung immune cells reclustered at a higher resolution to characterize the immune landscape, colored by cell type. **c**, UMAP visualization of 37,090 stromal lung cells reclustered at a higher resolution to characterize the stromal landscape, colored by cell type. **d**, Volcano plot showing top DE genes in alveolar macrophages in COVID-19 compared to LRTD with a significant adjusted *P* value (<0.05) and a log fold change of more than 0.5 using MAST followed by Bonferroni multiple test correction. **e**, Dotplot showing the average gene module score of IFN response pathways across alveolar macrophages in COVID-19 and LRTD. **f**, Violin plots showing the gene module score across alveolar macrophages in gene sets associated with the gamma, alpha, beta, lambda and IL6 response in COVID-19 compared to LRTD. Black lines indicate the mean value across all cells, with the log fold change between means across conditions annotated above the plots. gdT cell, gamma-delta T cell; NK, natural killer; NS, not significant; Treg, regulatory T.

To explore cellular responses in the lung at greater depth in Malawian patients, we used single-nuclei and single-cell RNA-seq in four patients with COVID-19, three patients with LRTD and one non-LRTD patient. Integrating 66,882 cells resulted in 16 cell clusters composed of a mixture of immune and stromal cells (Fig. 3a, Supplementary Fig. 2 and Supplementary Tables 5–7).

We detected few SARS-CoV-2 reads, suggesting that, at time of death, there was minimal replicating virus (Supplementary Fig. 4), consistent with our IMC data supporting inflammatory rather than direct viral-driven pathogenetic mechanisms.

**Extended Data Fig. 4:**
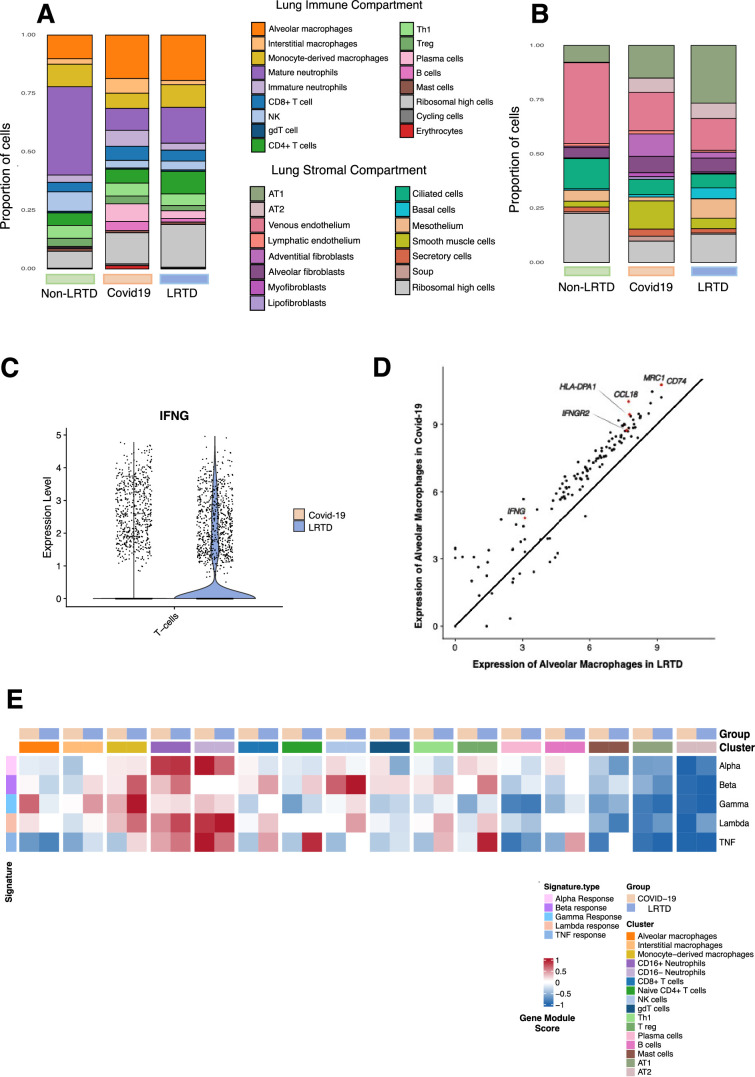
Lung cell proportions and gene module scores. (**a-b)** Cell type proportion bar plots of lung cell types in **(a)** Immune cells and **(b)** Stromal cells corresponding with Fig. 3b and c, grouped by disease group. (**c)** Violin plot to show a comparison between COVID-19 and LRTD cases of expression of IFNG (the IFN-γ gene) in a pseudo bulk analysis of lung scRNA-seq including all T-cells. **(d)** Plot shows expression levels of different IFNG module genes in lung alveolar macrophages between COVID-19 and LRTD cases. Line is at 1:1 ratio, hence dots to the left of the line indicate genes with higher expression in COVID-19 cases and to the right of the line indicates genes with higher expression in LRTD. Dots for genes for the IFN-γ receptors IFNGR1 and IFNGR2 and for IFNG (the IFN-γ gene) are indicated. **(e)** Heatmap showing the mean gene module score across cells in gene sets associated with the *alpha, beta, gamma, lambda and TNF response*. Cell types have been grouped by COVID-19 and LRTD to show the difference in response and module score values have been scaled between −1 and 1.

We then undertook finer annotation of immune cell (Fig. 3b) and stromal cell (Fig. 3c) pools. Consistent with IMC data, we identified alveolar, interstitial and monocyte-derived macrophages and mature and immature neutrophils. Stromal cells included adventitial and alveolar fibroblasts, type I and type II pneumocytes (AT1 and AT2) and basal, secretory and ciliated epithelial cells. Cell proportions should be interpreted with caution given the few patients per group, but they showed cell diversity expansion in the COVID-19 and LRTD groups not observed or absent in the LRTD group (Extended Data Fig. 4a,b).

Principal differences in COVID-19 compared to LRTD were in myeloid cells, particularly alveolar macrophages (Fig. 3d–f), with few differences in lymphocytes, dendritic cells or stromal cells (Supplementary Tables 6 and 7). In alveolar macrophages, top differentially regulated genes included markers of tissue residency (*C1QC* and *C1QB*)^35^ and factors shown to mediate lung fibrosis (*CCL18*)^36^ and apoptosis (*S1006*)^37^ and myeloid activation and recruitment (*SPP1*)^38^. Interferon-gamma (IFN-γ) response protein (*IFI30*) and major histocompatibility complex (MHC) proteins (*HLA-DRA* and *HLA-DRB1*) were all upregulated, indicating response to IFN-γ.

This IFN-γ-dominant response contrasts with type I and type III dominant IFN responses shown to be critical in pathogenesis in Northern Hemisphere COVID-19 cohorts^16,39^. Given the prominence of alveolar macrophages in the immune response and in alveolar damage identified by IMC, we analyzed alveolar macrophage IFN response pathways. IFN-γ modules were expressed in a high proportion of cells (Fig. 3e) and strongly upregulated in COVID-19 compared to LRTD (log fold change, 0.1136; Fig. 3f). IL6/JAK/STAT pathway was also expressed, but to a lower extent, and the difference from LRTD was less clear (log fold change, 0.0418). In contrast, IFN-α, IFN-β and IFN-λ were minimally expressed, without clear differences from LRTD. This increased IFN-γ response could be due either to increased IFN-γ production or to increased responsiveness in macrophages. Using a pseudobulk approach, *IFNG* (IFN-γ gene) in T cells was not different between patients with COVID-19 and patients with LRTD (Extended Data Fig. 4c). In contrast, IFN-γ response genes were consistently upregulated in alveolar macrophages (Extended Data Fig. 4d), together implying that the increased IFN-γ response in patients with COVID-19 is due to a heightened response propensity of lung-resident macrophages rather than simply a heightened inflammatory response. In support of this, across other myeloid cells, IFN responses were heterogeneous, and TNF response was upregulated in the LRTD group in several cell types (Extended Data Fig. 4e).

### Contrasting IFN responses between Malawian and other cohorts

**Fig. 4:**
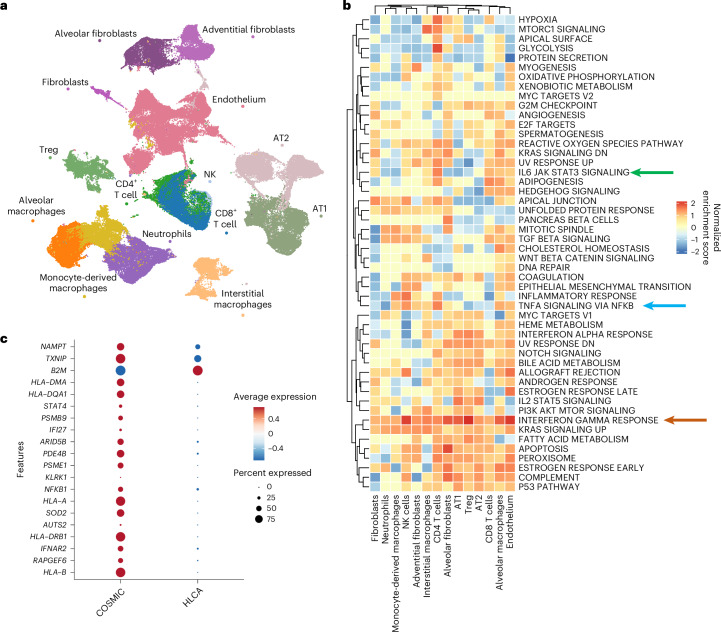
Integration with HLCA COVID-19 cohorts highlights dominant T cell macrophage IFN-γ axis in Malawian patients with COVID-19. **a**, UMAP visualization of 147,935 lung cells deriving from integrating cells from patients with COVID-19, patients with LRTD and non-LRTD patients from our cohort with cells from the HLCA from COVID-19, patients with LRTD and non-LRTD patients. Clusters are colored by cell type. **b**, Heatmap showing pathway analysis for DE genes in our COVID-19 cohort compared to the HLCA COVID-19 cohort. Shown are the 50 canonical hallmark gene sets (for list, see Supplementary Information) colored by the normalized enrichment score for each cell type. Gene Ontology pathways of interest are indicated by arrows (IL6 JAK STAT3 SIGNALING, green; TNFA SIGNALING VIA NFKB, blue; INTERFERON GAMMA RESPONSE, orange). **c**, Dotplot showing the average expression of top DE genes in the lung alveolar macrophages that contribute the highest in the hallmark gene set ‘INTERFERON GAMMA RESPONSE’ pathway in our COVID-19 cohort compared to the HLCA COVID-19 cohort. NK, natural killer; Treg, regulatory T.

To compare IFN responses with Northern Hemisphere cohorts, we integrated our Malawian single-cell data with multi-cohort COVID-19 (five cohorts, 60 patients), LRTD (one cohort, 13 patients) and non-LRTD (23 cohorts, 178 patients) data from the Human Lung Cell Atlas^10^ (HLCA) (Fig. 4a; cohorts summarized in Fig. 1b).

**Extended Data Fig. 5:**
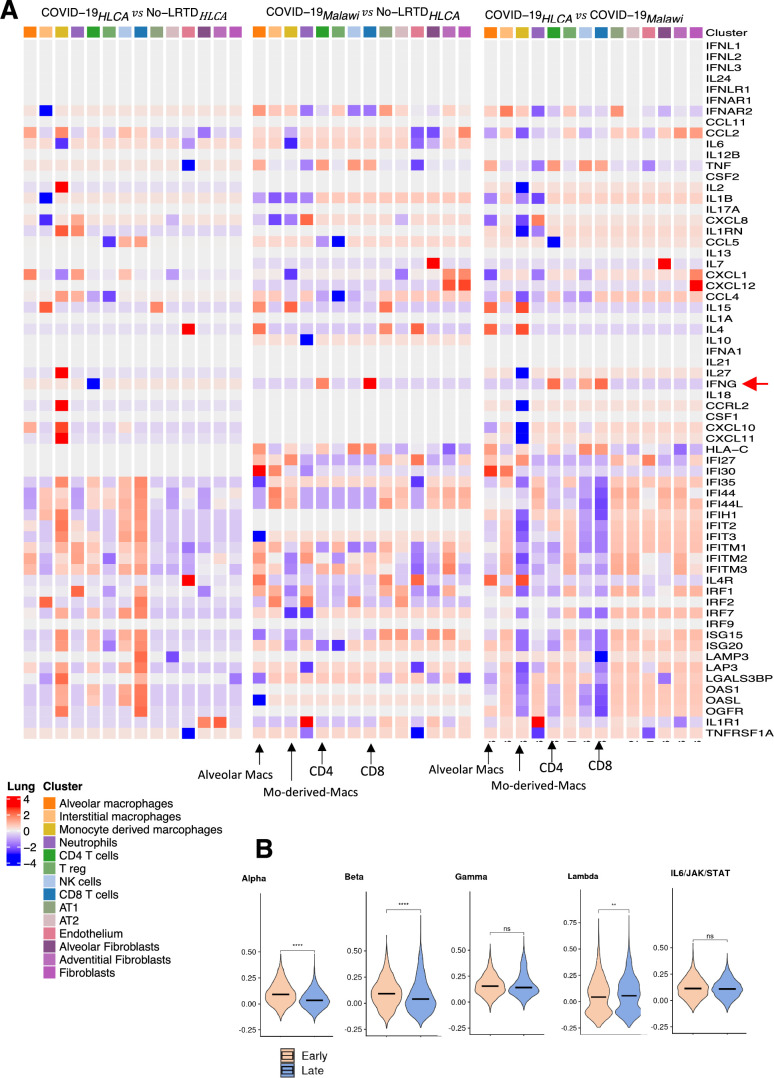
Heatmap of interferon response genes in lung. **(a)** Heatmaps showing the log fold change of up/down-regulated interferon response genes taken from immunologic gene sets involved in the immune response. Comparisons include the change in interferon response in cells from the HLCA COVID-19 cohort compared to HLCA control cases (left), the Malawi COVID-19 cohort compared to control cases from the HLCA (middle) and interferon responses from our COVID-19 cohort compared to the HLCA COVID-19 cohort (right). **(b)** Violin plots of IFN and IL6 response modules in lung tissue macrophages in USA cases (from Delorey et al) comparing early death cases that died within 2 weeks of illness onset (early) and late death cases that died after two weeks of illness (late). All p-values were calculated using a two-sample Wilcoxon test with Bonferroni multiple test correction (Alpha p < 2.2e-16 ****, Beta p < 2.2e-16 ****, Gamma p = 0.7141 ns, Lambda p = 0.09 **, IL6 p = 0.8939 ns).

Pathways indicative of IFN-γ response were increased across all cell types in the Malawian cohort (Fig. 4b, orange arrow) and were particularly upregulated in alveolar macrophages (Fig. 4c). Furthermore, *IFNG* (IFN-γ gene) was specifically increased in the Malawian cohort in CD4 and CD8 T cells versus HLCA COVID-19 and non-LRTD groups (Extended Data Fig. 5a). Other inflammatory pathways showed a mixture of upregulation and downregulation in the Malawian cohort compared to HLCA cohorts, including IL6/JAK/STAT (Fig. 4b, green arrow) and TNF-NFKB (Fig. 4b, blue arrow)—key targets for therapies being used in COVID-19. Many of the other IFN response genes were more upregulated in the HLCA cohorts or had a heterogenous distribution across cells, although, notably, monocyte-derived macrophages generally had a higher IFN response in HLCA COVID-19 cohorts (Extended Data Fig. 5a).

As with IMC data, we explored whether bias in illness duration explains differences in IFN responses among cohorts. Specifically, we compared IFN gene module scores in early versus late death patients in a US study with these metadata available. If the population-specific profiles were a function of illness stage, then, in the US cohort, we would expect higher IFN-γ levels in early death and higher IFN-α,β,λ levels in late death. Instead, IFN-α,β,λ responses were significantly stronger in early death, whereas the IFN-γ response was not different (Extended Data Fig. 5b).

Although many inflammatory pathways were shared between Malawian and Northern Hemisphere cohorts, the Malawian cohort exhibited amplified IFN-γ responses in lung-resident macrophages.

### Nasal cell responses may be a useful proxy for lung cell responses

Although the lung is the principal organ involved in severe COVID-19 disease, it would be useful to know if we can predict lung responses using nasal or blood samples that can readily be obtained during life.

**Fig. 5:**
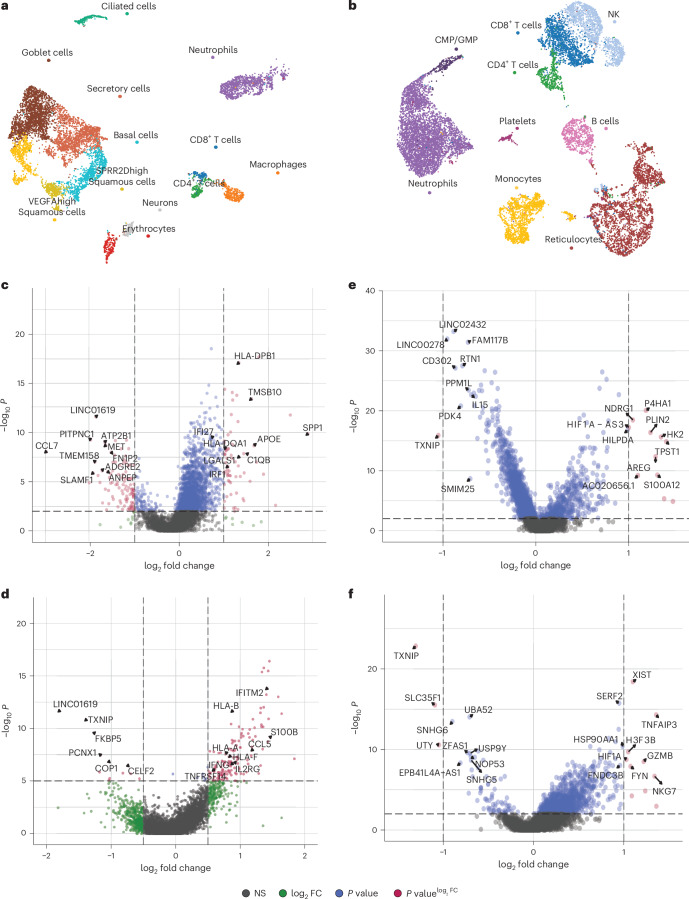
scRNA-seq of nasal and blood cells: nasal but not blood cells parallel lung IFN-γ response. **a**, UMAP visualization of 8,098 nasal cells across our cohort, colored by broad cell types. **b**, UMAP visualization of 13,350 peripheral blood cells across our cohort, colored by broad cell types. **c**,**d**, Volcano plots showing top DE genes in nasal macrophages (**c**) and T cells (**d**) in COVID-19 compared to LRTD with a significant adjusted *P* value (<0.05) and a log fold change of more than 0.5. **e**,**f**, Volcano plots showing top DE genes in peripheral blood monocytes (**e**) and CD4^+^ T cells (**f**) in COVID-19 compared to LRTD with a significant adjusted *P* value (<0.05) and a log fold change of more than 0.5. FC, fold change; NK, natural killer.

We performed scRNA-seq on nasal cells in eight patients (five COVID-19, two LRTD and one non-LRTD) and peripheral blood mononuclear cells (PBMCs) in seven patients (four COVID-19, two LRTD and one non-LRTD). We recovered 8,098 nasal cells that mapped to 10 clusters composing immune and stromal cells and 13,350 blood cells (Fig. 5a,b and Supplementary Fig. 3). Nasal macrophages had several differentially expressed (DE) genes in patients with COVID-19 versus patients with LRTD that mirrored alveolar macrophage responses (*SPP1*, *LGALS1* and *TMSB10*), including IFN-γ response genes (*HLA-DPB1*, *HLA-DQA1* and *C1QB*) (Fig. 5c). There was also *IFNG* (IFN-γ gene) upregulation in T cells in patients with COVID-19 (Fig. 5d). Pathway analysis also showed higher levels of IFN-γ response in macrophages and T cells (Fig. 5e). In blood, there was upregulation of inflammatory (*AREG*) and vascular damage (*NDRG1*) genes in COVID-19 in monocytes but no upregulation of *IFNG* or IFN-γ response genes (Fig. 5f). Hence, in our small cohort, nasal cells better paralleled lung response than blood cells, supporting previous COVID-19 (refs. ^40,41^) and non-COVID-19 (ref. ^42^) studies that highlighted the utility of nasal cells to predict respiratory immune responses.

We also assessed whether cytokine responses in plasma or nasal fluid could distinguish the inflammatory or IFN-γ response in patients with COVID-19 versus patients with LRTD. In nasal fluid but not blood, there was a non-significant trend toward several cytokines being higher in patients with COVID-19 than in patients with LRTD (Supplementary Fig. 5a,b). Using a pseudobulk approach in blood, nasal and lung cells, there was also no difference between *IFNG* or other cytokine genes between patients with COVID-19 and patients with LRTD (Supplementary Fig. 5c–e). Thus, IFN responses identified in single-cell data were not identified by bulk protein or transcriptomic approaches. This may reflect the greater discriminatory power of single-cell methods given small numbers in our study.

### Different myeloid interactions predict alveolar and vascular damage

**Fig. 6:**
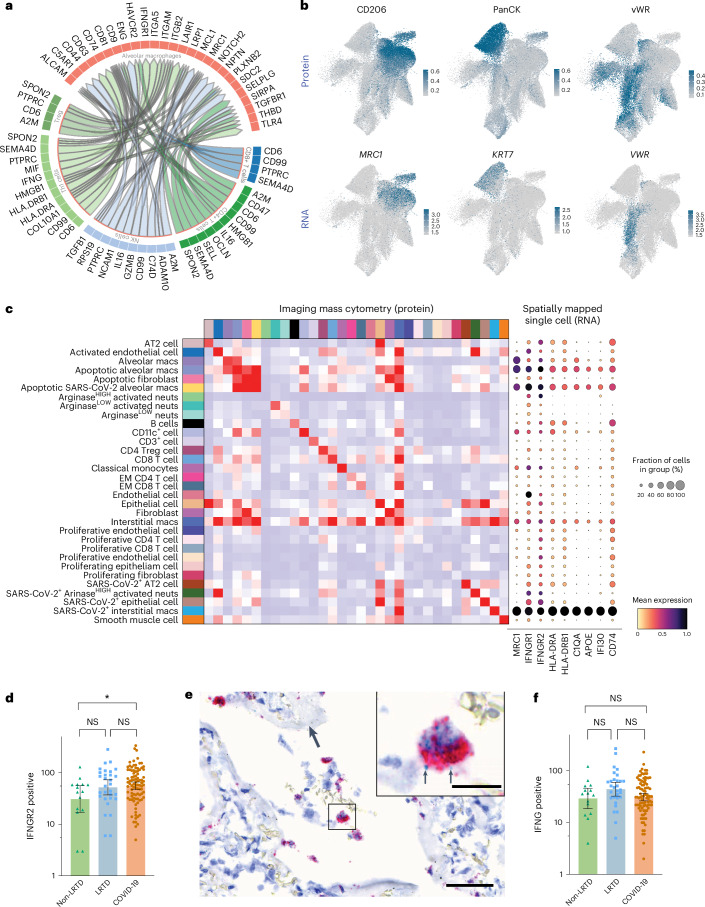
Spatially resolved cell interaction analysis predicts molecular mechanisms of alveolar and endothelial pathology. **a**, Circos plot showing the top cell–cell interactions from immune cells to alveolar macrophages in Malawian patients with COVID-19 versus Malawian patients with LRTD. Segments are colored by cell type with ligands and receptors labeled on the outside. Direction of the arrows shows the senders of communications that are expressing a given ligand to the receiver cell type expressing its cognate receptor. Inner tracks on sender segments are colored by the receiving cell type for ease of interpretation. **b**, UMAP plots to show expression levels of different hallmark proteins in different clusters by IMC and then below RNA levels from scRNA-seq data imputed by MaxFuse. **c**, Heatmaps showing co-localized cell types from IMC data, providing insight into potentially interacting cell types in the lung in patients with COVID-19; comparator LRTD and non-LRTD are in Extended Data Fig. 9. **d**–**f**, Quantification of mRNA in situ staining for IFNGR2 and IFNG in tissue. In total, 138 ROIs were taken based on multiple sampled areas from the left and right lung in nine patients with COVID-19, in three patients with LRTD and in two non-LRTD patients. Separate TMA sections were dual stained for either IFNGR2 and MRC1 (CD206) or IFNG and CD3E mRNA by in situ hybridization, and then the number of cells positive for each stain within respective cells of interest IFNGR2 in CD206^+^ cells and IFNG in CD3E^+^ cells was analyzed by automatic quantification. Each dot represents the quantities of positive cells in an independent tissue core that were used as replicates for analysis in **d** and **f**. These data were log transformed and analyzed using one-way ANOVA and Tukey’s multiple comparison test to adjust for multiple comparisons and a pre-defined alpha level of 0.05. Colored bars show the geometric mean, and error bars show the 95% confidence interval. **d**, Compared to non-LRTD patients, there were significantly higher numbers of IFNGR2^+^ cells in patients with COVID-19 but not in patients with LRTD (*P = 0.0441). **e**, Co-staining of IFNγR2 (red) and CD206 gene (green) using mRNA probes in lungs of patients infected with SARS-CoV-2. Lung of patients with COVID-19 shows, in the periphery of the damaged alveolar space fibrin (empty arrows) and in the lumen of the alveoli, cells with macrophage morphology expressing IFNGR2 (red signal, rectangle). The insert shows a higher magnification of the rectangle with a macrophage expressing CD206 in green (black arrows) and abundant IFNGR2 in red. Scale bars, 60 µm and 15 µm, respectively. Hematoxylin counterstaining. **f**, No significant difference was observed in quantities of IFNG^+^ cells among the different groups (NS, not significant; *P* = 0.111). EM, effector memory; macs, macrophages; neuts, neutrophils; Treg, regulatory T.

To assess the role of IFN-γ-responding resident macrophages in lung parenchymal pathology and neutrophil interactions in vascular pathology, and to predict molecular interactions for potential therapeutic targets, we used cell interaction methods. First, we performed unbiased receptor–ligand analysis of lung scRNA-seq data. A high proportion of predicted interactions involved lung-resident macrophages interacting with stromal cells and immune cells (Supplementary Fig. 6), fitting with findings from lesion analysis. We then did a targeted analysis of the interaction between T cells and alveolar macrophages, which identified a specific receptor–ligand interaction between *IFNG* from T helper cells (Th1) and IFN-γ receptor 1 (*IFNGR1*) on alveolar macrophages (Fig. 6a).

**Extended Data Fig. 6:**
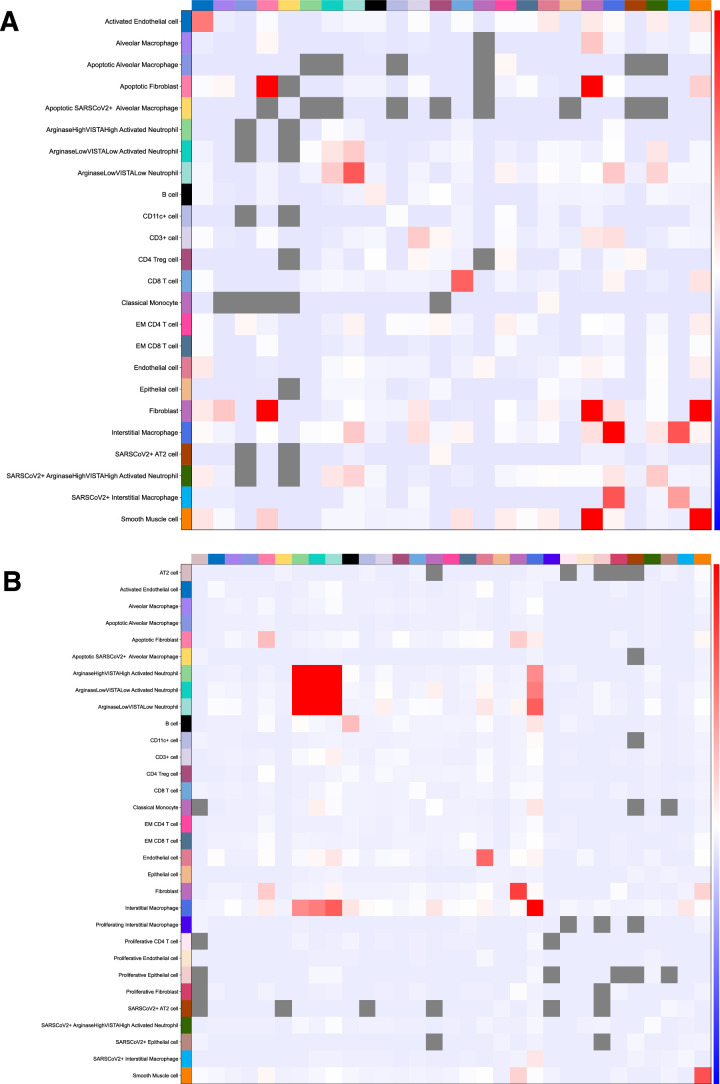
Predicted cell-cell interactions in the IMC datasets. Heatmaps for the non-LRTD group (**a**) and LRTD (**b**) showing co-localised cell types as shown by the IMC providing insight into potentially interacting cell types in the lung, shown for comparison with the same data from COVID-19 cases Fig. 6b (main figures).

**Extended Data Fig. 7:**
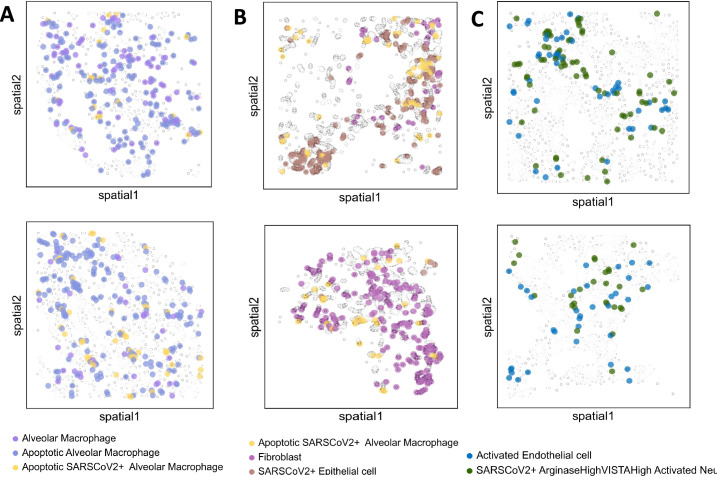
Visualisation of cell-cell interactions in the IMC datasets. Cellular maps showing the spatial location of specific immune cells – highlighting spatially enriched macrophage and neutrophil interactions identified in Fig. 6b (main figures). (**a**) shows interactions between alveolar macrophages (purple), apoptotic alveolar macrophages (blue) and apoptotic alveolar macrophages (yellow). **(b)** shows interactions between apoptotic alveolar macrophages (yellow) fibroblast (lilach) and SARSCoV2+ Epithelial cells (purple) (**c**) shows interactions between activated endothelial cells (blude) and SARSCoV2+ Neutrophils (green).

To validate these interactions in a spatial context in IMC, we conducted neighborhood enrichment analysis, which models cell proximity to predict likely cellular interactions. In the non-LRTD group, there were no significant interactions, whereas the LRTD group was completely dominated by neutrophil interactions (Extended Data Fig. 6a,b). In the COVID-19 group, several neighborhood enrichments were prominent—principally CD206^high^ alveolar macrophages (with and without SARS-CoV-2 and apoptosis) with stromal cells, apoptotic fibroblasts and type II pneumocytes (Fig. 6b, left, and Extended Data Fig. 7a,b). This supports a role in tissue damage of CD206^high^ macrophages. In contrast, the most prominent neighborhood enrichment for neutrophils was between SARS-CoV-2^+^, Arg^high^ neutrophils and activated endothelial cells, implicating neutrophils in vascular pathology (Fig. 6b, left, and Extended Data Fig. 7c).

**Extended Data Fig. 8:**
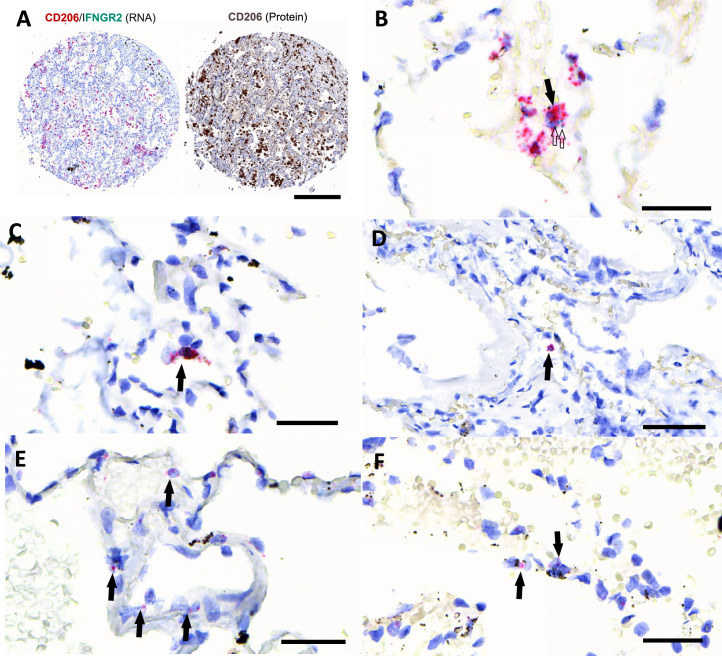
Dual in situ staining for CD206 and IFNGR2 (inducible IFN-γ receptor) and CD3 and IFNG (IFN-γ gene) for validation of IFN-γ response. 138 regions of interest were taken based on multiple sampled areas from the left and right lung in: 9 COVID-19 cases in 3 LRTD cases and 2 non-LRTD cases **a**) Shows adjacent sections of cores from a COVID-19 (Cos009) case to demonstrate concordance of CD206 RNA and protein staining. The left has had in situ staining (MRC1/CD206 in red and IFNGR2 in green) and the right image shows immunohistochemistry using an anti-CD206 antibody (staining in brown), bar 300μm. **b**) In situ staining for CD206 and IFNGR2 in LRTD case (Cos004); few cells are present with co-staining of IFNGR2 (green, arrow) and CD206 (red, empty arrow), bar 30μm. **c**) In situ staining for CD206 and IFNGR2 for non-LRTD case (Cos 016) only single positive cells are detected in general expressing IFNGR2 (green, arrow) as well as CD206, bar 30μm. **d**) In situ staining for IFNG and CD3 in a LRTD case (Cos003), CD3 red signal arrows, no green signal for IFNG has been detected, bar 60μm. **e**) LRTD case (Cos011), CD3, red signal, single positive cells, no co-staining with IFNG, arrows, bar 30μm. **f**) Non-LRTD cases (Cos016), CD3 in red, black pigment is interpreted as anthracosis, bar 30μm.

To spatially resolve this IFN-γ response in Malawian patients, we integrated scRNA-seq and IMC data and mapped gene expression profiles onto IMC cells using a recently developed pipeline^43^ (Fig. 6b). The integrated output showed upregulation of IFN-γ response genes, including *HLA-DR*, *IFI30* and *APOE*, and the inducible component of the IFN-γ receptor (*IFNGR2*) in tissue-resident CD206^high^ alveolar and interstitial macrophages (Fig. 6c, right). Notably, the IFN-γ response was most prominent in the SARS-CoV-2^+^ and apoptotic CD206^high^ macrophage populations, predicted to interact with apoptotic fibroblasts and type II pneumocytes in the neighborhood analysis (Fig. 6c, left). Thus, mapping scRNA-seq data onto our IMC data not only validates the IFN-y response but also implicates these IFN-γ-responding cells in lung stromal cell damage. Additionally, in situ hybridization staining across patients and 138 ROIs also highlighted significantly higher numbers of *IFNGR2*^+^ cells in patients with COVID-19 than in non-LRTD controls but not between non-LRTD patients and patients with LRTD (Fig. 6d and Extended Data Fig. 8a–f). *IFNGR2* was predominantly in CD206^high^ cells, which could be observed in diffuse alveolar damage lesions (Fig. 6e). In contrast, the number of *IFNG*^+^ cells was not significantly increased in patients with COVID-19 (Fig. 6f), validating findings from scRNA-seq (Extended Data Fig. 4c). Thus, multiple orthogonal methods demonstrate an IFN-γ response in CD206^high^ lung-resident macrophages, and this is best explained by the responsiveness of these cells rather than increased inflammation and *IFNG* production.

**Extended Data Fig. 9:**
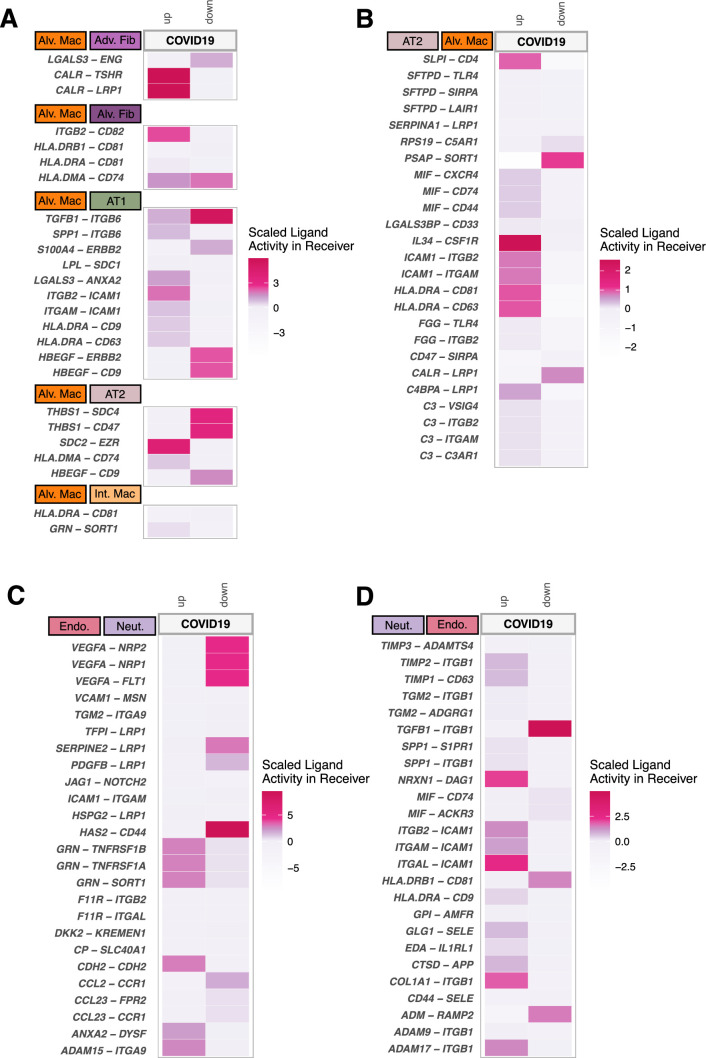
Predicted receptor ligand interactions in single cell data. **a**) Heatmap showing up/down-regulated interactions in COVID-19 compared to LRTD driven by AT2 pneumonocytes to alveolar macrophages. Coloured boxes indicate cell type with the ligand-expressing cell type followed by the receptor-expressing cell type. **b**) Heatmap showing up/down-regulated interactions in COVID-19 compared to LRTD driven by lung alveolar macrophages to lung epithelial cells and interstitial macrophages. Coloured boxes indicate cell type with the ligand-expressing cell type followed by the receptor-expressing cell type. **c**) Heatmap showing up/down-regulated interactions in COVID-19 compared to LRTD driven by lung endothelium to neutrophils. Coloured boxes indicate cell type with the ligand-expressing cell type followed by the receptor-expressing cell type. **d**) Heatmap showing up/down-regulated interactions in COVID-19 compared to LRTD driven by neutrophils to lung endothelium. Coloured boxes indicate cell type with the ligand-expressing cell type followed by the receptor-expressing cell type.

We then looked at validated interactions in COVID-19 in closer detail in scRNA-seq data to predict interactions that might indicate therapeutic targets. Macrophage interactions were frequently with type II pneumocytes (Extended Data Fig. 9a,b), in keeping with type II pneumocytes cells being a principal infected cell^44^. Several of these interactions involved macrophage inhibitory factor (*MIF*) from type II pneumocytes with *CD74*, *CD44* and *CXCR4* on macrophages, a classical response chain in macrophages and a key initiator of proliferation, chemotaxis and activation^45^. *ICAM-1* on type II pneumocytes was predicted to signal to integrins (*ITGB2-ITGAM*) on alveolar macrophages, an interaction involved in cellular attachment during recruitment. Another strong predicted interaction was *IL-34-CSF1R*, involved in triggering macrophage activation and chemotaxis. Reciprocally, there were several interactions between alveolar macrophages and epithelial cells that support their role in alveolar pathology, consistent with IMC data. These included *SPP1* and *TGFB1* with integrin (*ITGB6*) in AT1 cells (Extended Data Fig. 9a), interactions implicated in lung pathology and fibrosis^38,46,47^. We identified multiple neutrophil interactions with endothelial cells, indicating processes involved in neutrophil attachment to the vascular wall (for example, *ITGAL-ICAM-1*) and of activation by neutrophil granule proteins (*GRN-TNFRSF1A*) (Extended Data Fig. 9c,d), providing molecular validation supporting their role in coagulation, endothelial activation and vascular pathology, as suggested by analysis of lesions using IMC.

## Discussion

We conducted a postmortem study and characterized pulmonary, blood and nasal immune responses in COVID-19 using histology, scRNA-seq and high-dimensional imaging in a Malawian population. We initially hypothesized that an attenuated immune response to SARS-CoV-2 in SSA populations might lead to high lung viral burden and, thus, to severe disease being the consequence of direct viral effects. This would indicate a need for different treatment approaches from Northern Hemisphere cohorts where hyperinflammation is predominant. Reassuringly, instead, we found a robust immune response, comparatively low levels of virus and many histopathological and immunological similarities to non-African cohorts, even in immunosuppressed patients with HIV. However, there were also differences that may have implications for therapy. We identified a dominant IFN-γ response in lung-resident macrophages, increased in comparison to a large multi-country integrated HLCA dataset. Spatially resolved interaction analysis and scRNA-seq receptor–ligand analysis implicated these IFN-γ-responding resident macrophages in lung damage. In contrast, IL6, TNF and type I/III IFN responses were not as prominent as in other cohorts.

There is crossover among the responses of different IFNs, yet this dominant IFN-γ response is noteworthy in this context as prior infection exposures, including malaria, have been shown to induce augmented IFN-γ response^17^, specifically through epigenetic changes, termed trained immunity^18^, and IFN-γ, enhanced by prior Bacille Calmette-Guérin (BCG) exposure, has been implicated in clearance of SARS-CoV-2 infection^48^. Such responses may be a double-edged sword in COVID-19, being generally protective (through more rapid viral clearance) but, in a subset of patients, leading to accelerated hyperinflammation and collateral tissue damage. To test the specific hypothesis that malaria, or other infections, is the driver of these different immune responses in COVID-19 in SSA populations would require larger studies of both SSA and non-SSA populations with different levels of exposure to these infections.

Considering the potential for immediate translation, existing therapies for COVID-19 target JAK/STAT (baricitinib), IL6 (toculimazab/sarilumab) or TNF (infliximab)^15,16^. JAK/STAT signaling is a conserved pathway for IFN responses, including IFN-γ^49^. Thus, our data, if corroborated, support potential efficacy of baricitinib over other treatments. Baricitinib is a small molecule (tablet) and, thus, highly suited to wide distribution^15^.

Our data have several limitations. Our cohort is small and in a single center. We could not fully control for all variables, leaving the cause of different immune responses uncertain, including a potential impact of different viral variants. Although single-cell methods have a higher capacity to resolve complex data in small sample sizes, many analyses in our study are underpowered. It is, thus, unclear how representative our data are of the wider Malawian or other SSA populations. Studies in other settings, and, ideally, large multi-center studies, are needed. Although this would be a complex undertaking, we have demonstrated that single-cell methods are feasible in an SSA setting, and our study provides useful templates. Although lung samples cannot readily be obtained in live patients, postmortem studies have limitations: cells may change or degrade, and pathological processes present early in disease are likely missed. However, postmortem studies in Northern Hemisphere settings with longer postmortem intervals identified validated targets^7^. Although minimally invasive autopsy is more feasible and acceptable than traditional open autopsy, blinded sampling may attenuate the identification and sampling of areas of pathology. However, except for large airway pathology, which was not sampled, most COVID-19 features were identified. The studies that we used for comparisons had considerable variation in methods and demographics from ours, which may induce noise and bias. We used data integration methods that reduce, but do not eliminate, these. Reassuringly, findings were validated both by comparison to other cohorts and by orthogonal IMC data and targeted in situ staining.

Our data highlight the value of a combined scRNA-seq and high-dimensional imaging approach. They provide spatial and receptor–ligand validation for a role of IFN-γ-responding tissue-resident macrophages in alveolar damage and for neutrophils in endothelial activation. The data highlight specific molecular interactions involved in these processes. If validated by further work, some of these interactions may highlight additional plausible targets for intervention—for example, *MIF*, for which several small molecules are in clinical development for therapy in inflammatory disorders^45^. Our de-identified data, provided open access and through visualization tools, make an important resource for furthering the global understanding of COVID-19 pathogenesis and immune responses in SSA populations, as part of the HCA.

## Methods

### Ethics

This study complies with all relevant ethical regulations. The protocol for the Malawian study was approved by the National Health Scientific Research Committee in Malawi (protocol number 07/09/1913) and by the Medical Veterinary Life Sciences ethics committee in Glasgow (protocol number 200190041). The study protocol for the Brazilan study was approved by the local research ethics committee at Tropical Medicine Foundation Dr. Heitor Vieira Dourado, Manaus, Western Brazilian Amazon (protocol numbers CAAE:30152620.1.0000.0005 and CAAE:32077020.6.0000.0005). Additional studies on this cohort were published separately^13,50^. We also used open-access de-identified IMC data from a published US-based autopsy study conducted at New York Presbyterian/Weill Cornell Medicine Hospital, for which the study protocol was approved by the institutional review board at Weill Cornell Medical College^34^. Informed consent was taken from the families of deceased patients for all patients at all sites.

### Patients

We recruited patients aged 45–75 years who were admitted to QECH, Blantyre, between October 2020 and July 2021, during which there were two epidemiological waves driven by different SARS-CoV-2 variants: Beta (December 2020–February 2021) and Delta (May–July 2021)^41^. Patients admitted with respiratory signs were routinely tested for SARS-CoV-2 at QECH. We recruited patients into three groups based on clinical criteria: (1) a COVID-19 group (*n* = 9) with clinical features suggesting acute respiratory distress (ARDS, oxygen requirement and respiratory signs on either clinical examination or chest X-ray changes or both) and who had at least one nasal swab positive for SARS-CoV-2 on admission; (2) a non-COVID-19 LRTD group (*n* = 5) with clinical signs of ARDS but negative for SARS-CoV-2 on admission and during hospitalization; and (3) a no-LRTD, COVID-19-negative group (*n* = 2) with no oxygen requirement and no clinical signs of LRTD and for which the admission and any subsequent nasal swabs were negative for SARS-CoV-2 on polymerase chain reaction (PCR) (Fig. 1b and Extended Data Table 1). Clinical, premortem and postmortem laboratory data were entered into REDCap; double entry was used and checked by a third investigator, with discrepant results resolved by consulting the original source. The study only recruited patients who died between 24:00 and 12:00 to minimize the postmortem interval and to avoid doing any autopsies at night. None of the patients included had received any SARS-CoV-2 vaccine; only approximately 2% of the Malawian population had received a first dose by study completion.

### Minimally invasive autopsy

We used minimally invasive tissue sampling (MITS) to conduct autopsies with large-bore needle biopsies of organ samples rather than full autopsy^23^. Being more culturally acceptable, MITS is widely used to determine cause of death in pediatric studies^23–25^, showing good concordance with full autopsy^24^. From our ongoing pediatric MITS studies in Malawi, we adapted protocols for adult patients with COVID-19 to obtain tissue suitable for scRNA-seq and IMC, based on the protocol from the Child Health and Mortality Prevention Surveillance (CHAMPS) network but with adaptations. A larger-caliber needle (11 gauge) was used for biopsies to obtain larger tissue samples. Samples were taken from the brain through supraorbital sampling from both left and right sides. From each lung, samples were taken from lower-middle and upper zones from a single entry point, angling the needle to sample different areas. Nasal cells were collected from the nasal inferior turbinate using curettes (ASL Rhino-Pro, Arlington Scientific). Two curettes were collected from each nostril, and the cells were placed immediately into ice-cold HypoThermosol (STEMCELL Technologies). Cells were transported on ice in a cold box immediately to the laboratory and were spun at 300*g* for 5 min for either immediate processing for scRNA-seq or storage in a CryoStor 10 (see below). Nasal fluid was collected using matrix strips (Nasosorption, Hunt Developments). One strip was used per nostril. Personal protective equipment (PPE) was worn by all staff involved in the autopsies and for all work in the laboratory. Laboratory work on samples was performed in vented laminar flow hoods.

### Processing and storage of samples

Biopsies from each organ were collected in three different ways for different downstream workflows: (1) for paraffin embedding for histology and IMC, put in 10% neutral buffered formalin; (2) for viable cells, put in ice-cold HypoThermosol (STEMCELL Technologies) for transport to the laboratory and then slow freeze in a CryoStor 10 (STEMCELL Technologies); and (3) for snap-frozen cells, put in cryovials and then seal and immediately submerge in liquid nitrogen.

Biopsies were fixed in 10% neutral buffered formalin for 4–8 h, rinsed in water and then embedded in paraffin blocks. Samples for viable cells were rinsed and cut into pieces of approximately 20–50 mm and then put into ice-cold CryoStor for 15–30 min before transfer to a −80 °C freezer in a chilled cryogenic storage container (CoolCell, Corning).

Blood cells collected into sodium heparin tubes were separated from plasma by spinning at 400*g* for 10 min. Plasma was then removed and spun for an additional 10 min at 1,500*g*, and plasma was frozen in aliquots at −80 °C. Cells were resuspended in 10% FBS in PBS, and PBMCs were separated using Ficoll-Paque with a 27-min spin at 450*g* and either used immediately for scRNA-seq or pelleted and resuspended in ice-cold CryoStor 10 and then moved to a −80 °C freezer in a chilled cryogenic storage container (CoolCell, Corning). The next day, samples were moved from the −80 °C freezer to liquid nitrogen for long-term storage. Snap-frozen samples were transferred in a liquid nitrogen dewar and then moved to liquid nitrogen storage tanks for long-term storage.

### Pathology and organ-specific scoring

Formalin-fixed tissues were paraffin embedded (FFPE) for lung, bone marrow, brain, spleen and liver to make blocks. FFPE blocks were sectioned at 2–4-μm thickness, mounted on glass slides and stained with H&E. A medical pathologist (S.K.) reviewed tissue slides, alongside patient histories and antemortem laboratory results per standard clinical practice, and completed an organ-specific scoring proforma that included COVID-19 features (Supplementary Table 3). Then, for a non-biased assessment, two additional pathologists, blinded to diagnosis, scored the lung pathology in all patients using systematic scoring criteria. Lung tissue was scored independently by two additional pathologists (C.A. and V.H.) who were blinded to patient history and previous diagnoses. After individual scoring, any discrepancies were discussed by joint review of the slides until a consensus was reached. The lung scoring was semi-quantitative for the parameters indicated in Extended Data Fig. 1a–c. Subsequently, we characterized each sample with a dominant histological characteristic—for example, fibrinopurulent inflammation/pneumonia in case the neutrophil infiltration with fibrin extravasation was marked next to a mild infiltrate of lymphocytes, plasma cells and macrophages. Whole-tissue slides from lung samples in our nine patients with COVID-19 can be accessed in their entirety and visualized at various magnifications, as if they were observed under a microscope, using our virtual microscope tool: https://covid-atlas.cvr.gla.ac.uk (de-identified slides will be uploaded and publicly viewable upon publication).

After scoring, in each lung biopsy, the most representative areas were manually selected based on the scoring performed on the H&E-stained section to create the TMAs with cores of 1 mm in diameter using the TMA Grand Master (3DHISTECH) and CaseViewer software (version 2.4.0119028). At least eight ROIs were taken from each case (four left, four right). From the newly created TMA-FFPE blocks, 4-mm-thick sections were cut and used for downstream IMC, in situ hybridization or bright-field immunohistochemistry.

### Cause of death attribution

A panel consisting of the pathologist who reviewed the patients, respiratory physician, intensive care physician, infectious disease physician and two trainee doctors reviewed all the patients to assign a cause of death. Codes assigning death were given according to International Classification of Diseases (ICD) codes and using the standard coding system used for death certification. The review consisted of a review of the clinical notes, premortem and postmortem laboratory results and the pathology report. Each member reviewed the documents independently and reached an individual verdict. When there were discrepancies, a consensus was reached through discussion.

### Multiparameter cytokine assay

Cytokine levels were measured in plasma and nasal fluid samples using Luminex with the Inflammation 20-Plex Human ProcartaPlex panel (Thermo Fisher Scientific, EPX200-12185-901) according to the manufacturer’s protocol and levels measured with a Luminex MagPix device. Data were transformed with a log_2_ and for the visualization with ComplexHeatmap in R with a z-score by cytokine.

### IMC

Sections from TMAs underwent deparaffinization, followed by antigen retrieval at 96 °C for 30 min in Tris-EDTA at pH 8.5. Non-specific binding was blocked with 3% BSA for 45 min, followed by incubation with lanthanide-conjugated primary antibodies (overnight at 4 °C), which were diluted in PBS with 0.5% BSA (Supplementary Information). Antibodies were conjugated with metals using Maxpar Antibody Labeling Kits (Standard BioTools) and were validated with positive control tissue (tonsil and spleen for immune-targeted antibodies). Slides were then washed with 0.1% Triton X-100 in PBS, followed by nuclear staining with iridium (1:400; Intercalator-Ir, Standard Bio Tools) for 30 min at room temperature and, finally, briefly (10 s) washed with ultrapure water and air dried. Images were acquired on a Hyperion imaging mass cytometer as per the manufacturer’s instructions (Standard BioTools). Each TMA core was imaged in a separate ROI.

### IMC analysis

Pre-processing, imaging denoise, cell segmentation and extraction of single-cell features were performed using a combination of Python and R packages, including ImcSegmentationPipeline, IMC-Denoise^51^ and DeepCell^13,34,52^. For the single-cell analysis, the annotated data object was generated, and protein expression raw measurements were normalized at the 99th percentile to remove outliers. In Scanpy (version 1.9.1), principal component analysis (PCA), batch correction and Harmony data integration were performed to compute and plot the uniform manifold approximation and projection (UMAP) embeddings (umap-learn Python package, version 0.5.3). Next, automated cell type assignment using the Python package Astir (version 0.1.4) was applied to identify the major cell types expected to be found in the lung tissue according to the antibody panel used. For cell assignment with Astir, the following information to label cells based on a broad ontogeny (metaclusters and major cell types) and the proteins (lineage markers) to be most expressed in each expected cell type were used: (1) macrophage: CD163, CD206, CD14, CD16, CD68, CD11c, Iba1; (2) neutrophil: CD66b, Arginase1; (3) CD8 T cells: CD3, CD8; (4) CD4 T cells: CD3, CD4; (5) B cells: CD20; (6) endothelium: CD31; (7) fibroblast: Collagen1; (8) SMC: smooth muscle actin; epithelial: PanCK; RBCs: CD235ab.

After cell assignment, cells labeled as ‘other’ or ‘unknown’ were filtered out from downstream analysis, and the annotated data object was subset into the major cell types identified—that is, macrophages, neutrophils, lymphoid, vascular, epithelial and stromal—and Phenograph Louvain clustering (with 200 nearest neighbors) was performed for each cell population separately using a small set of specific lineage marker and functional proteins. The finer cell type annotation was used to evaluate the frequency and absolute counts of cell types across clinical groups, histopathological lesions and HIV status. Differential abundance analysis was also performed using the scanpro and scCODA Python packages^53^ and the miloR R package (version 1.4.0)^54^. Spatial statistics analysis based on the coordinates of the cells in the ROIs was performed using the Python package Squidpy (version 1.2.2)^55^. These coordinates were used to plot spatial graphs and to calculate and plot neighborhood enrichment scores^13^.

### Integration of Malawian IMC data with other available IMC COVID-19 lung data

IMC COVID-19 data from postmortem lung samples from published Brazilian^13^ and US^34^ fatal cohorts were integrated with the Malawian IMC dataset. First, datasets were concatenated in Scanpy taking the ‘inner’ (intersection) of all common protein markers in the panels across the three IMC datasets. Then, with scvi-tools^56^, we applied different integration methods, such as Harmony and variational autoencoder (VAE)-based methods, such as scVI and scANVI. Analysis of the UMAP embedding of the integrated versus non-integrated data showed that Harmony and scANVI performed better, and, in downstream analysis, we used Harmony-integrated output. Next, cell identities were standardized (label harmonization), which refers to a process of checking that labels are consistent across the datasets that are being integrated. Finally, cell frequencies in the postmortem lung across all three cohorts were plotted, and differential abundance analysis was performed using scanpro (https://github.com/loosolab/scanpro) and scCODA Python packages^57^ and the miloR R package (version 1.4.0)^58^.

### Dissociation of lung cells from frozen samples and single-nuclei preparation

Lung samples were dissociated both from fresh samples and from slow-frozen samples that had been stored in liquid nitrogen. Slow-frozen cells were defrosted in a water bath at 37 °C, and then pieces of tissue were transferred to RPMI 1640 medium with 25 mM HEPES and L-glutamine (Thermo Fisher Scientific) and 40% heat-inactivated FBS (Thermo Fisher Scientfic). Fresh or defrosted frozen cells were then dissociated, adapting a previously published protocol for lung dissociation^57^. Samples were dissociated in a buffer containing 400 mg ml^−1^ Liberase DL (Sigma-Aldrich), 32 U ml^−1^ DNAse I (Roche) and 1.5% BSA in PBS (without calcium and magnesium). The tissue was put in buffer (four times weight:volume) in a GentleMACS C-tube (Miltenyi Biotec, 130-096-334), minced using scissors and then run on a GentleMACS dissociator (Miltenyi Biotec, 130-093-235) on the manufacturerʼs program ‘C-lung 01_02’. Dissociation was achieved by warming the tissue on an orbital shaker in a chamber at 37 °C for 30 min and running ‘C-lung 01_02’ twice more: once at 15 min and once at 30 min. The enzyme was neutralized by diluting with 10 ml of ice-cold 20% FBS, containing 32 U ml^−1^ DNase. The sample was then filtered through a 100-µm strainer (Corning, 352360), and samples were subsequently kept on ice with all centrifuge and antibody incubation steps at 4 °C. Cells were pelleted by spinning at 300*g* for 5 min at 4 °C. RBCs were removed by incubating with ACK lysing buffer (Thermo Fisher Scientific, A1049201) for 5 min at room temperature. For frozen cells, debris and dead cells were removed using a debris removal solution (Miltenyi Biotec, 130-109-398) and a dead cell removal kit (Miltenyi Biotec, 130-090-101), respectively, according to the manufacturerʼs protocol.

Single nuclei were isolated from snap-frozen lung tissue samples using a previously published method^7^. Tissue was kept on dry ice/liquid nitrogen until processing was started. Tissue was placed into a GentleMACS C-tube containing 2 ml of freshly prepared nuclei extraction buffer that contained RNAse inhibitors: 0.2 U µl^−1^ RNaseIN Plus RNAse inhibitor (Promega) and 0.1 U µl^−1^ SUPERasin RNAse inhibitor (Thermo Fisher Scientific). Dissociation was achieved by running the C-tube on the GentleMACS dissociator on program ‘m_spleen_01’ for 1 min. The sample was filtered using a 40-µm strainer and spun at 500*g* for 10 min at 4 °C. Pellet was then resuspended in 500 µl of 1× ST without RNAse inhibitor and filtered again using a 35-µm strainer. A 10-µl volume was loaded on a hemocytometer for counting.

### Single-cell and single-nuclei partitioning and library preparation

10x 3′ v3 chemistry was used for all samples. For fresh lung samples, we loaded 10,000 cells into one channel of a 10x chip (1000120). For fresh nasal and blood samples, we labeled the nasal and blood samples with different hashtags and pooled them at a 1:1 ratio and loaded 10,000–20,000 cells. For frozen nuclei and single-cell samples, we pooled samples from 3–6 different patients aiming for equal ratios and loaded 20,000–40,000 cells per nuclei. Libraries were prepared according to the manufacturer’s protocol and sequenced with an Illumina NextSeq 2000. To make these data available for analysis by others, reads were submitted to ArrayExpress (E-MTAB-13544).

### Single-cell data processing

For all tissue compartments, the data were analyzed through the following steps. (1) Processing of the raw reads. 5′ scRNA-seq data along with the 3′ snRNA-seq runs were demultiplexed using Cell Ranger ‘mkfastq’. Reads were mapped to a concatenated human GRCh38, SARS-CoV-2 (severe acute respiratory syndrome coronavirus 2 isolate Wuhan-Hu-1, GenBank MN908947.3) and HIV (human immunodeficiency virus 1, GenBank AF033819.3) reference genome to generate count matrices using Cell Ranger ‘cellranger count’ (version 7.0). (2) Ambient RNA removal. To reduce potential noise driven from empty droplets or ambient RNA captured in our samples, we used the tool SoupX (version 1.6.2)^58^ and used corrected expression matrices in subsequent analyses. (3) Quality control and filtering. Data were analyzed using the Seurat package (version 4.3)^59^ in R (version 4.2) with mitochondrial gene expression thresholding applied on individual samples. In addition, cells that were expressing more than 150 genes were retained to maximize discovery of cell types. (4) Normalization and variance stabilization. Samples were merged and normalized using the SCTransform() function, selecting the top 3,000 variable genes to drive the downstream clustering. Additionally, effects of mitochondrial gene expression, ribosomal gene expression and cell cycle were regressed out. (5) Integration. PCA was run on all merged data objects. The embeddings were then fed into the standard Harmony (version 0.1.1)^56^ integration pipeline. (6) Clustering and dimensionality reduction. An appropriate number of principal components (PCs) were selected to generate the UMAP. PCs were used to determine the *k*-nearest neighbors for each cell for the shared nearest neighbor (SNN) graph construction, followed by clustering at resolution 0.3. (7) Cell type annotation. Identification of cluster markers for the lung and nasal datasets were calculated by running FindAllMarkers() using MAST, followed by Bonferroni multiple test correction. We specified that genes must be expressed in at least 25% of cells (min.pct = 0.25) with a log fold change of 0.25. Cell types were manually annotated, leveraging canonical cell type markers reported from existing literature and curated datasets. Peripheral blood clusters were annotated using the consensus label transfer algorithm SingleR (version 2.0.0)^60^ using the Azimuth Reference PBMC atlas (https://zenodo.org/records/4546839). Cells with low mapping scores were reanalyzed and manually annotated as above. (8) Gene Ontology (GO) and pathway analysis. DE genes across conditions were calculated using the FindMarkers() function using MAST. Genes were defined as DE with a significance threshold of less than 0.05 and a log fold change threshold of 0.25, followed by Bonferroni correction. Gene set enrichment analysis (GSEA) was done using the fgsea package (1.3.0)^61^ using 50 canonical hallmark gene sets as described in the Molecular Signatures Database (MSigDB) (version 7.5.1)^62^. (9) Module scoring. Gene module scoring was calculated using the AddModuleScore() function of gene sets taken from MSigDB and AmiGO 2 (ref. ^63^) that related to IFN responses (lambda (GO:0034342), alpha (GO:0035455), IFN-β (GO:0035456), IFN-γ (GO:0034341), IL6/JAK/STAT (*HALLMARK_IL6_JAK_STAT3_SIGNALING*) and TNF (*HALLMARK_TNFA_SIGNALING_VIA_NFKB*)). log fold changes in module scores were calculated using the log2 + 1 of the differential means across a cell type. (10) Cell–cell communication analysis. Inference of cellular communications was computed using the multinichenetR (version 1.0.3) package^64^ with a log fold change cutoff of 0.5 being expressed in at least 10% of cells across conditions.

### Hashtag demultiplexing

Hashtag reads were quantified using CITE-seq-Count (version 1.4.4)^65^ and demultiplexed using cellHashR (version 1.0.1)^66^. The following methods were tested: BFF_cluster_, BFF_raw_ (10), GMM-Demux^67^, Seurat HTODemux^59^ and DropletUtils hashedDrops^68^, with HTODemux resulting in the highest number of singlets that were used for analysis.

### Single-nucleotide polymorphism splitting of multiplexed runs

Demultiplexing of runs was carried out using the single-nucleotide polymorphism (SNP) clustering algorithm Souporcell^69^ to identify distinct genotypes and assign cells to different individuals. For each run, we set the number of clusters (*k*) to the expected number of genotypes in the run (*k* = 2–6), and cell barcodes were assigned to each cluster. Cluster barcodes were then used to subset the input BAM file across human leukocyte antigen (HLA) loci of the multiplexed runs, under the assumption that these would be distinct regions of the genome for each individual. Using Integrative Genomics Viewer (IGV), we visualized SNP distributions at a set allele frequency of 0.2 and compared the subset BAM files to BAM files from individual runs. Iteratively, Souporcell clusters were assigned to samples through the following rationale: (1) matching SNP distributions to independent sequencing runs, (2) through mapping to sex chromosomes or (3) through the process of elimination where an independent sequencing run genotype was not available. In scenarios where Souporcell failed to identify the expected number of genomes, we assigned cluster barcodes to matching genotypes from independent sample runs regardless of expected *k*. After successful demultiplexing, we identified which cells derived from which patient and were able to proceed with downstream single-cell analyses as outlined above (see ‘Single-cell data processing’ subsection).

### HLCA integration

The HLCA^10^ was filtered down, retaining cells that were taken from the lung and lung parenchyma. These included studies originating from the Northern Hemisphere, with lung cell data in COVID-19, pneumonia and healthy controls. Cell type annotations harmonized with our analyses (*AT1*, *AT2*, *EC arterial*, *EC capillary*, *EC venous*, *Fibroblasts*, *Innate lymphoid cell*, *NK*, *Macrophages*, *Monocytes*, *T cell lineage*) were selected. To have sufficient power for downstream analyses with our cohort, we randomly subsampled each cell type within each disease condition to create a normalized atlas of 100,000 cells to integrate with our lung atlas. Processing and integration steps were followed as described previously for the Malawian cohort using 38 PCs and a clustering resolution of 0.2. Manual cluster annotation was performed by running FindAllMarkers(), leveraging canonical cell type markers.

### Pseudobulking single-cell nasal and blood

To make our nasal and blood scRNA-seq comparable with Luminex cytokine data, we assigned all cells to a unified identifier (‘pseudo_cluster’) to pool cells belonging from different cell type clusters together. Then, the average expression of the different cytokines on the Luminex panel were visualized using ComplexHeatmap^70^ and a z-score of the counts (Supplementary Fig. 5). For the statistical tests of genes associated with the IFN-y pathway, we used a Welch two-sample *t*-test.

### Exploring viral reads in samples

To identify SARS-CoV-2-infected cells in our lung dataset, we quantified the number of unique molecular identifiers (UMIs) that were detected after mapping with Cell Ranger across our single-cell datasets. A given cell was deemed to be infected if it expressed at least two UMIs of genes mapping to the SARS-CoV-2 genome.

### Integration of Malawian COVID-19 lung IMC data with Malawian COVID-19 lung snRNA-seq data

Lung IMC and snRNA-seq data, exclusively from Malawian patients with COVID-19, were integrated with the recently developed integration tool MaxFuse, which integrates data across weakly linked modalities, such as protein and RNA expression, through cross-modality matching and iterative smoothed embedding^43^. Highly variable features (s.d. > 0.3 for the RNA expression and s.d. > 0.1 for the protein expression) shared between both datasets were retrieved based on a protein-to-gene correspondence list, produced by the MaxFuse authors and edited to include specific protein markers in our IMC panel (Supplementary Information). Cell counts used for each modality included IMC (53,762 cells) and snRNA-seq (36,616 cells). Previously normalized and batch-corrected IMC protein expression and snRNA-seq RNA expression were used as MaxFuse input. All values were capped between 5% and 95% quantiles for visualization purposes. With the resulting integration, expression levels of IFN-γ response-related genes (*IFNGR1*, *IFNGR2*, *HLA-DRA*, *HLA-DRB1*, *C1QA*, *APOE*, *IFI30* and *CD74*) and IFN-γ signature score were determined and plotted in the lung cells derived from the IMC data.

### In situ hybridization co-staining for *CD3* and *IFNG* and *CD206* (*MRC1*) and *IFNGR2*

In situ staining was performed on TMAs with 138 ROIs using the same TMAs and patients used for IMC, covering multiple lung regions from left and right lungs in nine patients with COVID-19, three patients with LRTD and two non-LRTD patients. Consecutive slides were used for two dual staining panels: one for *IFNG* and *CD3E* and the other for *IFNGR2* and *MRC1* (CD206). Slides were stained according to the manufacturer’s instructions (product codes: 322452 and 322500, ACD, Bio Techne) using the probes Hs IFNG-C1, Hs IFNGR2-C1, Hs-MRC1-C2 and Hs CD3E-C2 (product codes: 310501, 553971-C2, 1269501-C1 and 583921-C2, ACD, Bio Techne) and positive and negative control probes PPIB/POLR2A and DapB (product codes: 321641 and 320751, ACD Bio Techne). Slides were digitized and scanned with standard settings at ×80 magnification using the Motic EasyScan Infinity 60 digital slide scanner (I. Miller Microscopes). For quantification of positive cells, we used HALO software (version 3.6.4134.362) with the AI module (3.6.4134) and the FISH module (version 3.2.3) for cell detection after deconvolution.

### Immunohistochemistry

Immunohistochemistry was performed in an autostainer using the Envision kit and DAB chromogen (product codes: K4003 and K4001, Agilent Technologies) with anti-CD206/MRC1 (E2L9N) or anti-CD3 antibodies (product codes: 91992, Cell Signaling Technologies, and A0452, Agilent Technologies). Slides were digitized and scanned at ×20 magnification using an Aperio VERSA 8 slide scanner (Leica Biosystems) and Aperio VERSA 1.0.4.125 software (Leica Biosystems).

### Statistics and reproducibility

No statistical method was used to predetermine sample size. We excluded nine single-cell sequencing runs that had few to no cells and that did not pass standard quality control metrics. Within our lung atlas, a population of cells (*n* = 1,348) was excluded that we deemed to be low-quality cells that almost exclusively derived from one multiplexed single-nuclei sequencing run that exhibited extremely low UMI counts. Two non-COVID-19 patients with LRTD were excluded from IMC runs as they had evidence of active TB lung disease because of theoretical safety concerns, as IMC can generate aerosol. Pathologists were blinded to patient groups for systematic scoring of the lung, and investigators conducting the in situ validation experiments undertook staining and automated scoring on the TMAs blinded to which samples were from which case or group. For other experiments and analyses, investigators were not blinded to case groups. Samples were sequenced as multiplex, including patients from different groups, and IMC was run on TMAs as a single run, in both instances to reduce batch effect.

### Ethics and inclusion statement

Malawian researchers with clinical, laboratory, analysis and medical ethics expertise were involved throughout the research process from conception to manuscript preparation. The main research questions were determined by Malawian clinical and laboratory researchers alongside international researchers who were living and working in Malawi. Before conducting the study, we undertook a full sensitization process for the study with all staff on the recruiting wards in our hospital to discuss the study and consider the best way of sensitively conducting recruitment and informed consent. This work was led by two social scientists (L.S. and D.N.), one specialized in bioethics (D.N.). Details of our approach and considerations for recruitment are published as a chapter in a casebook separately^71^. Extensive research and laboratory infrastructure already exists in Malawi through a medical university (Kamuzu University of Health Sciences) and several internationally funded research programs. Building on this, as part of this project, local research capacity was enhanced by establishing a single-cell platform in Malawi and training local scientists and by additional training of local scientists in tissue processing. As a result, all tissue processing and cell partitioning and library preparation for single-cell and single-nuclei sequencing were done in Malawi. The research protocol was approved by a Malawian research ethics review committee (National Health Service Research Ethics Committee) and, in the United Kingdom, by the University of Glasgow Medicine Veterinary and Life Sciences Research Ethics Committee. Safety of staff was ensured by conducting renovations to create a dedicated autopsy room for COVID-19 autopsies and by providing PPE and cleaning solutions and training all staff that were patient facing or involved in sample collection and handling in their appropriate use. Laboratory work was conducted in a laminar flow hood using PPE. Local and regional research, including autopsy studies and investigative work, were considered throughout the study and are appropriately cited.

### Reporting summary

Further information on research design is available in the Nature Portfolio Reporting Summary linked to this article.

## Online content

Any methods, additional references, Nature Portfolio reporting summaries, source data, extended data, supplementary information, acknowledgements, peer review information; details of author contributions and competing interests; and statements of data and code availability are available at 10.1038/s41591-024-03354-3.

## Supplementary information

Supplementary Information

Reporting Summary

Supplementary Table 1 Additional clinical information.

Supplementary Table 2 Antemortem and postmortem laboratory results.

Supplementary Table 3 Histopathology scoring for different organs. Each organ is in a different tab: lung, spleen, brain, bone marrow, liver and heart.

Supplementary Table 4 Comparison of cell proportions in IMC data. The table is divided into different sections by tabs of the Excel file. The clinical groups Malawian tab shows the proportion of different immune, stromal and vascular cells in the three groups in the Malawian cohort: COVID-19, LRTD and non-LRTD and statistical comparison among the groups. The HIV tabs shows comparison between HIV-positive and HIV-negative COVID-19 cases in the Malawian cohort. The cohorts tab shows comparison among the three IMC cohorts after integration: Brazilian, US and Malawian. The cohorts and progression tab shows these same comparisons in integrated data, but the cases in each cohort are divided into subgroups based on whether patients died within the first 14 d after illness onset (early death) or more than 14 d after illness onset (late death). This division is made only for the Brazilian and US cohorts as the Malawian cohort had only one case that died after 14 d. The cohorts and variants tab shows these same comparisons in integrated data but where the cases in the Malawian cohort are divided into subgroups based on the relevant viral variant; US and Brazilian cases are not subdivided because all cases were ancestral variant.

Supplementary Table 5 Summary statistics of Cell Ranger output and processing of scRNA/snRNA-seq data. The table is organized into three tabs detailing the mapping summary statistics for each scRNA/snRNA-seq run included in the study, quatification and demultiplexing statistics for the hashtagged runs and the summary statistics for the SNP splitting genotype assignment.

Supplementary Table 6 Cell counts for single-cell data for lung, nasal and blood cells from the Malawian cohort split by disease group. Each tab shows cell counts for different immune and stromal cell types for COVID-19, LRTD and non-LRTD cases.

Supplementary Table 7 Differential gene expression in single-cell data by cell type. Differential gene expression analysis results from all cell types in the lung, nasal and blood tissue compartments. The table is organized with each comparison of cell types in the Malawian cohort in COVID-19 compared to LRTD in the three tissues and includes comparisons between lung cells in the Malawian cohort compared to the HLCA cohort. The table contains the average log fold change (avg_log_2_FC) along with the *P* value (p_val) and multiple test corrected *P* values (p_val_adj).

## Figures and Tables

**Extended Data Table 1:**
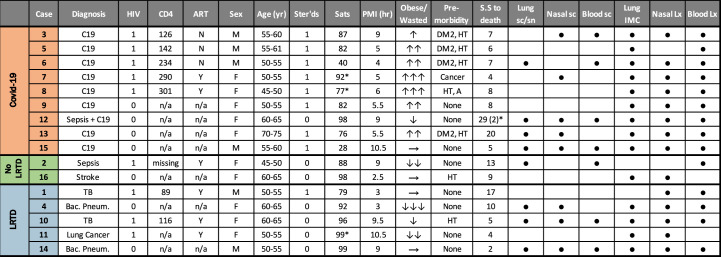
Characteristics of the patients

Summary table of cases recruited into our study.

PMI, postmortem interval in hours.

Obese/Underweight indicates nutritional status, determined by a combination of abdominal circumference measurements and mid-arm circumference measurements and based on reference data for men and women in African populations: ↑, overweight; ↑↑, obese; ↑↑↑, morbidly obese; ↓, underweight; ↓↓, severely underweight.

Pre-morbidity: DM2, type 2 diabetes mellitus; HT, hypertension.

S.S. to death, symptom start to death, indicating the number of days between the first symptoms consistent with COVID-19 (fever, cough, headache, etc.) and death.

Lung IMC, imaging mass cytometry; Lung sc, lung cell single-cell RNA-seq; Nasal sc, nasal cell single-cell RNA-seq; Blood sc, blood cell single-cell RNA-seq; Nasal Lx, nasal Luminex, for multiplexed cytokine array on nasal fluid. A dot for each of these parameters indicates that data are available for that assay for that case.

## Data Availability

scRNA-seq: Raw data and processed count matrices are deposited at the EBI ArrayExpress (accession number E-MTAB-13544). Fully processed RDS objects of the scRNA-seq analysis and IMC can be found through the GitHub repository (https://github.com/olympiahardy/COSMIC_Malawi_Covid_Atlas) and through the following Zenodo records: 10.5281/zenodo.13898422 (ref. ^72^) and 10.5281/zenodo.13899297 (ref. ^73^). The atlases are browsable using the Cellxgene VIP platform hosted by the University of Glasgow at the following URLs: Lung Atlas: https://cellatlas-cxg.mvls.gla.ac.uk/COSMIC/view/COSMIC_Lung_Atlas.h5ad/ Lung Immune Atlas: https://cellatlas-cxg.mvls.gla.ac.uk/COSMIC/view/COSMIC_Lung_Immune_Atlas.h5ad/ Lung Stromal Atlas: https://cellatlas-cxg.mvls.gla.ac.uk/COSMIC/view/COSMIC_Lung_Stromal_Atlas.h5ad/ Nasal Atlas: https://cellatlas-cxg.mvls.gla.ac.uk/COSMIC/view/COSMIC_Nasal_Atlas.h5ad/ Blood Atlas: https://cellatlas-cxg.mvls.gla.ac.uk/COSMIC/view/COSMIC_Blood_Atlas.h5ad/ Histopathology slides on virtual microscope: https://covid-atlas.cvr.gla.ac.uk Metadata for the patients (without identifying information) are provided in Extended Data Table 1 and Supplementary Tables 1 and 2. IMC: https://cellatlas-cxg.mvls.gla.ac.uk/COSMIC/view/COSMIC_IMC_Lung.h5ad/

## Code availability

All R scripts for the scRNA-seq analysis and figure generation can found at https://github.com/olympiahardy/COSMIC_Malawi_Covid_Atlas. Python scripts to process the imaging mass cytometry and figure generation can be found at https://github.com/joaolsf/Spatial_Single_Cell_Lung_Atlas_Malawi_COVID.
