# Supplementary material for: Spatially resolved single-cell atlas unveils a distinct cellular signature of fatal lung COVID-19 in a Malawian population
Source: Nat Med. 2024 Nov 20;30(12):3765–77. doi: 10.1038/s41591-024-03354-3 (PMC11645280; doi:10.1038/s41591-024-03354-3)
Supplement: Supplementary file 1 — Supplementary Information [file 41591_2024_3354_MOESM1_ESM.pdf]

nature medicine

Supplementary information https://doi.org/10.1038/s41591-024-03354-3

Spatially resolved single-cell atlas unveils a distinct cellular signature of fatal lung COVID-19 in a Malawian population

In the format provided by the authors and unedited

Supplementary Information for:

Spatially resolved single-cell atlas unveils a distinct cellular signature of fatal lung COVID-19 in a Malawian population

Nyirenda et al.

Table of contents

Gene panel in IFN-γ response 2

Antibody panel clones and catalogue numbers for imaging mass cytometry 3

Table of software packages used 4

Supplemental Figure 1 7

Supplemental Figure 2 8

Supplemental Figure 3 9

Supplemental Figure 4 10

Supplemental Figure 5 11

Supplemental Figure 6 12

Supplemental Figure 7 13

Supplemental Figure 8 14

Figure Legends – Supplemental Figures 15

Software References 17

Gene panel defining the IFN-γ response

To investigate the various interferon responses we used genes that are associated with each gene ontology term (see Single cell processing section). In particular we examined the interferon gamma response which were defined with the following 125 genes: *CD74, TLR2, CCL16, TLR3, CCL25, SHFL, CAMK2A, CALCOCO2, HPX, SYNCRIP, CDC42, ADAMTS13, IFITM2, IFITM3, ACTR2, ACTR3, STXBP4, SIRPA, SLC26A6, MEFV, RAF1, GBP7, CCL26, IL23R, WAS, IL12RB1, GBP6, CASP1, IL12B, KYNU, CCL14, CALM1, GBP2, GBP1, MRC1, TYK2, CD58, ASS1, DAPK3, CD47, GCH1, RAB7B, SLC11A1, SNCA, NUB1, RAB20, STAT1, CCL3, CD40, IRF1, CXCL16, CLDN1, FLNB, XCL2, EDN1, CDC42EP4, CCL15, CCL3, L1GSN, CCL22, GAPDH, CX3CL1, STXBP1, STXBP3, LGALS9, CCL24, RAB43, CCL19, KIF5B, WNT5A, MYO1C, TP53, GBP3, IFITM1, CCL11, ACTG1, TNFSLC30A8, FASLG, CCL20, VAMP3, CCL17, CCL7, IFNGR2, SLC22A5, CCL8, BST2, CCL13, PDE12, DAPK1, XCL1, CITED1, ZYX, CIITA, IFNG, AQP4, CCL21, AIF1, CDC42EP2, CCL5, CCL2, STX4, IRF8, JAK2, HLA-DPA1, STX8, RPL13A, IFNGR1, TRIM21, CYP27B1, GBP5, GBP4, VIM, HCK, VPS26B, CCL4, UBD, ACOD1, CCL18, CCL2, 3NOS2, TLR4, SP100, JAK1, RPS6KB1*.

Antibody panel for imaging mass cytometry

ANTIBODY	SOURCE	IDENTIFIER
rabbit anti-SARS-CoV-2 nucleocapsid protein (5ug/ml)	Novus Biologicals	Cat# NB100-56576
rabbit anti-human CD31 (2ug/ml)	Novus Biologicals	Cat# NB100-2284
rabbit anti-human SP-C (4.17ug/ml)	Novus Biologicals	Cat# NBP1-60117
mouse anti-human CD68 (5ug/ml)	Agilent	Cat# M0876
rabbit anti-human KRT8 (2.5ug/ml)	Abcam	Cat# ab59400
anti-human Smooth muscle actin (SMA) (clone 1A4)	Bio-Rad	Cat# MCA5781GA
anti-humanCD68 (clone KP1)	Thermo	Cat# MA5-13324
anti-human CD235ab (clone HIR2)	BioLegend	Cat# 306615
anti-human Pan-cytokeratin (PanCK) (clone AE-1/AE-3)	Biolegend	Cat# 914204
anti-human CD38 (clone EPR4106)	Standard Biotools	Cat# 3141018D
anti-human MHC I (clone EMR8-5)	Abcam	Cat# ab70328
anti-human Vimentin (clone RV202)	Standard Biotools	Cat# 3143029D
anti-human CD14 (clone D7A2T)	Cell Signalling	Cat# 56082BF
anti-human ICAM1 (clone EP1442Y)	Abcam	Cat# ab271852
anti-human CD16 (clone SP175)	Abcam	Cat# ab243925-
anti-human iNOS (clone SP126)	abcam	Cat# ab239990
anti-human CD66b (G10F5)	Novus	Cat# g10f5_nb100-
anti-human CD11b (EP1345Y)	Abcam	Cat# ab52478
anti-human CD44 (IM7)	BioLegend	Cat# 103001
anti-human CD107a (H4A3)	Standard Biotools	Cat# 3151021D
anti-human CD45 (CD45-2B11)	eBioscience	Cat# 14-9457-82
anti-human CD31 (JC/70A)	Novus	Cat# jc-70a_nb600-
anti-human CD11c (Polyclonal)	Standard Biotools	Cat# 3154025D
anti-human Foxp3 (clone 236A/E7)	Abcam	Cat# ab20034
anti-human CD4 (clone EPR6855)	Standard Biotools	Cat# 3156033D
anti-human SARS-Cov-2 (Polyclonal)	Novus	Cat# NB100-56576
anti-human Von Willebrand Factor (vWF) (Polyclonal)	Dako	Cat# A0082
anti-human Vista (clone D1L2G)	Standard Biotools	Cat# 3160025D
anti-human CD20 (clone H1)	Standard Biotools	Cat# 3161029D
anti-human CD8 (clone CD8/144B)	eBioscience	Cat# 14-0085-82
anti-human Iba1 (Polyclonal)	WAKO	Cat# 019-19741
anti-human Arginase 1 (clone D4E3M)	Standard Biotools	Cat# 3164027D
anti-human Fibrinogen (EPR18145-84)	Abcam	Cat# ab227063
anti-human CD74 (clone LN2)	Standard Biotools	Cat# 3166025D
anti-human Granzyme B (clone EPR20129-217)	Standard Biotools	Cat# 3167021D
anti-human Collagen Type I (Polyclonal)	Standard Biotools	Cat# 3169023D
anti-human CD3 (clone D7A6E)	Cell Signalling	Cat# 85061BF
anti-human pERK1/2 [T202/Y204] (clone D13.14.4E)	Standard Biotools	Cat# 3171021D
anti-human Cleaved Caspase 3 (clone 5A1E)	Standard Biotools	Cat# 3172023A
anti-human CD45RO (clone UCHL1)	Standard Biotools	Cat# 3173016D
anti-human MHC II (clone TAL1B5)	Abcam	Cat# ab176408
anti-human CD206 (clone E2L9N)	Cell Signalling	Cat# 91992
anti-human CD163 (clone EDHu-1)	Bio-Rad	Cat# MCA1853

Software packages used

Package name	Reference (where applicable)	URL
R v4.3.0		https://www.cran.r-project.org
Python v3.9.6		https://www.python.org
RStudio software (v 2023.03.0+386; 2023)	The R project for Statistical Computing	https://www.rstudio.com/ and https://cran.r-project.org/
Ilastik v1.4.0	Berg et al., 2019	https://www.ilastik.org
CellProfiler v4.2.4		https://cellprofiler.org
FactoMineR v2.8 (R package)	Sebastien Le, 2008	https://cran.r-project.org/web/packages/FactoMineR/index.html
ggplot2 v3.4.2 (R package)		https://ggplot2.tidyverse.org
factoextra v1.0.7 (R package)		https://cran.r-project.org/web/packages/factoextra/index.html
corrplot v0.92 (R package)		https://cran.r-project.org/web/packages/corrplot/vignettes/corrplot-intro.html
missMDA v1.18 (R package)	Josse and Husson, 2016	https://cran.r-project.org/web/packages/missMDA/index.html
tidymodels v1.0.0 (R package)		https://www.tidymodels.org
cytomapper v1.9.2 (R package)	Nils Eling, 2020	https://github.com/BodenmillerGroup/cytomapper
miloR v1.4.0 (R package)	(Dann et al., 2022)	https://marionilab.github.io/miloR
Giotto v3.3.0 (R package)	Dries et al., 2021	https://giottosuite.readthedocs.io/en/latest/
ImcSegmentationPipeline (Python package)	Vito RT Zanotelli, 2022	https://github.com/BodenmillerGroup/ImcSegmentationPipeline
IMC-Denoise (Python package)	Lu et al., 2023	https://github.com/PENGLU-WashU/IMC_Denoise
DeepCell (Python package)	Greenwald et al., 2022	https://github.com/vanvalenlab/deepcell-tf

Scanpy (Single-Cell Analysis in Python) v1.9.1 (Python package)	(Wolf et al., 2018	https://github.com/scverse/scanpy
umap-learn v0.5.3 (Python package)	(McInnes, 2018	https://github.com/lmcinnes/umap
Astir (ASsignmenT of sIngle-cell pRoteomics) v0.1.4 (Python package)	Geuenich et al., 2021	https://github.com/camlab-bioml/astir
Squidpy (Spatial Quantification of Molecular Data in Python) v1.2.2 (Python package)	Palla et al., 2022	https://squidpy.readthedocs.io/en/stable/
Athena (Analysis of Tumor HEterogeNeity from spAtial omics measurements) v 0.1.3 (Python package)	(Martinelli and Rapsomaniki, 2022	https://ai4scr.github.io/ATHENA/index.html
SpOOx (Spatial Omics Oxford Pipeline) (Python package)	(Praveen Weeratunga, 2022	https://github.com/Taylor-CCB-Group/SpOOx

Supplemental Figure 1 | Histological characteristics of lung pathology in patients with SARS CoV-2 infection

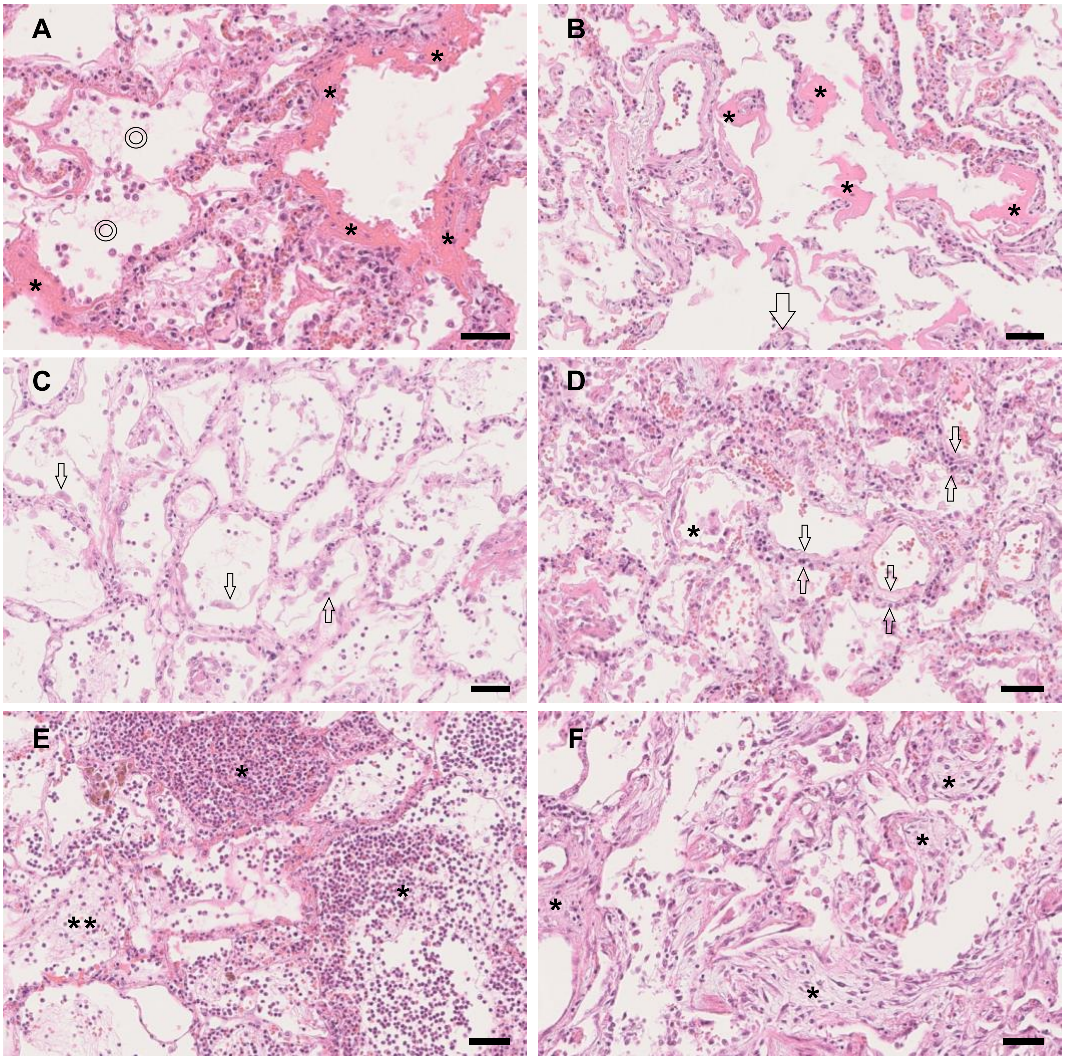

Supplemental Figure 2 | Top cluster markers characterising immune and stromal cell populations in the lung in the Malawi cohort

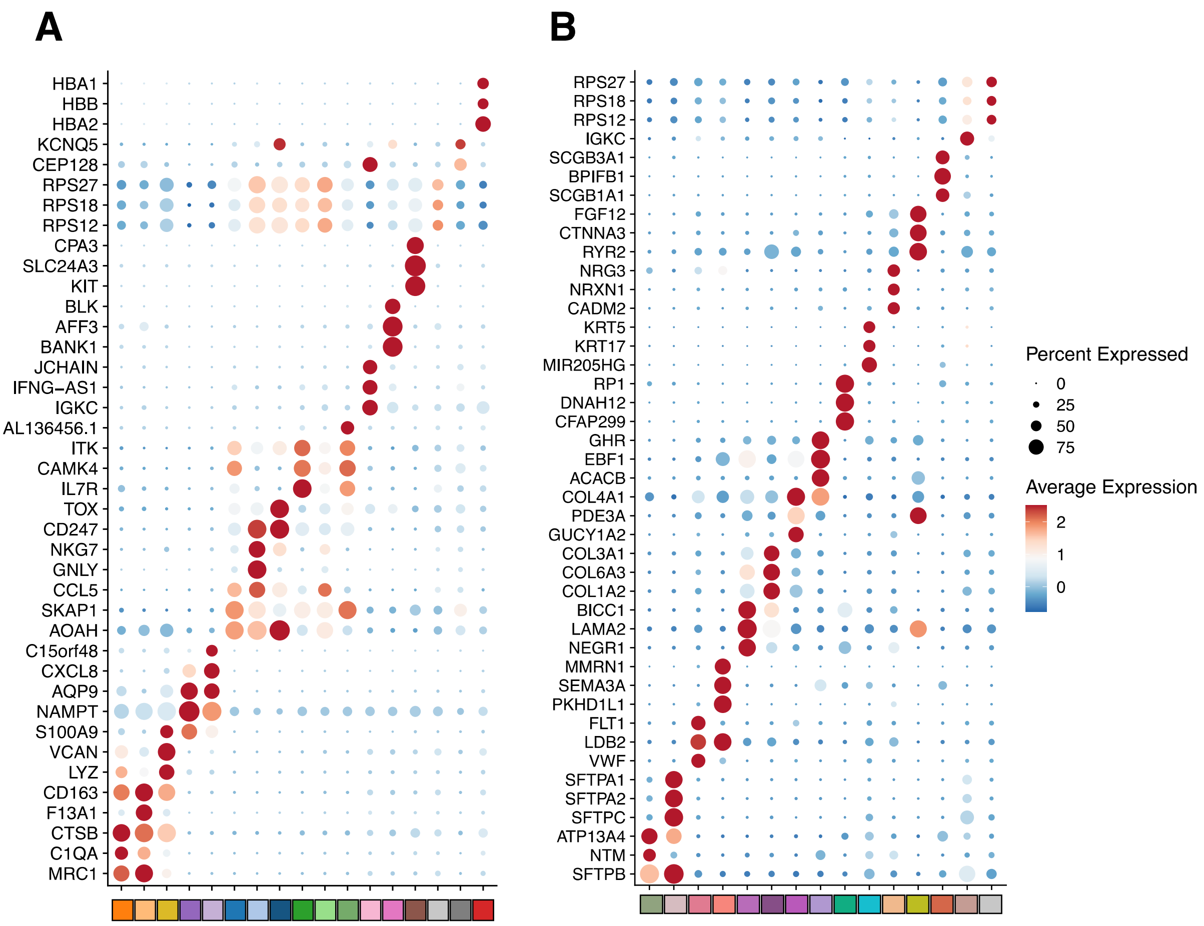

Supplemental Figure 3 | Characterisation of cell populations in the nasal and peripheral blood tissue compartments in the Malawi cohort.

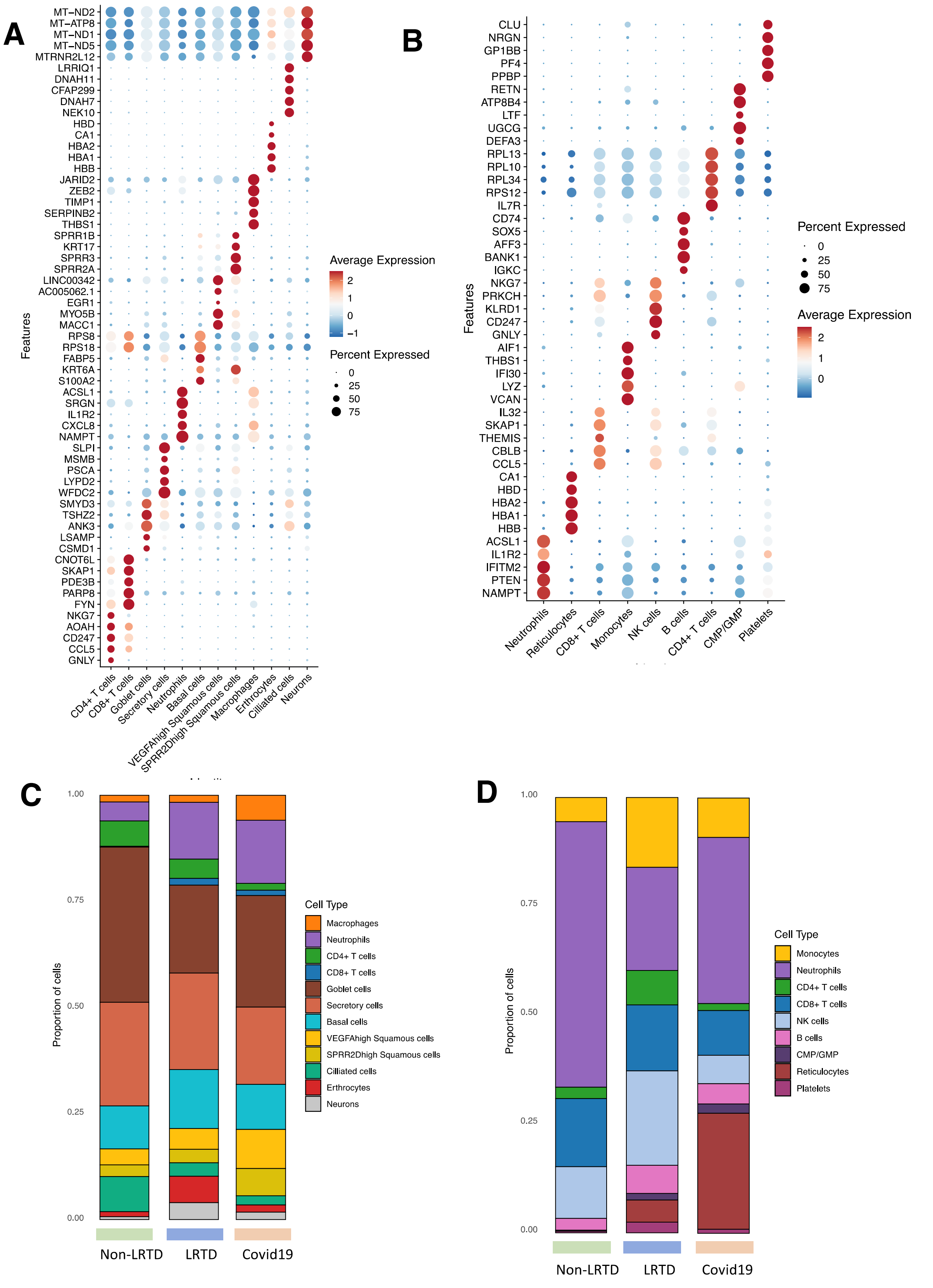

Supplemental Figure 4 | Minimal SARS-CoV2 reads in single-cell data of lung, nasal and blood cells

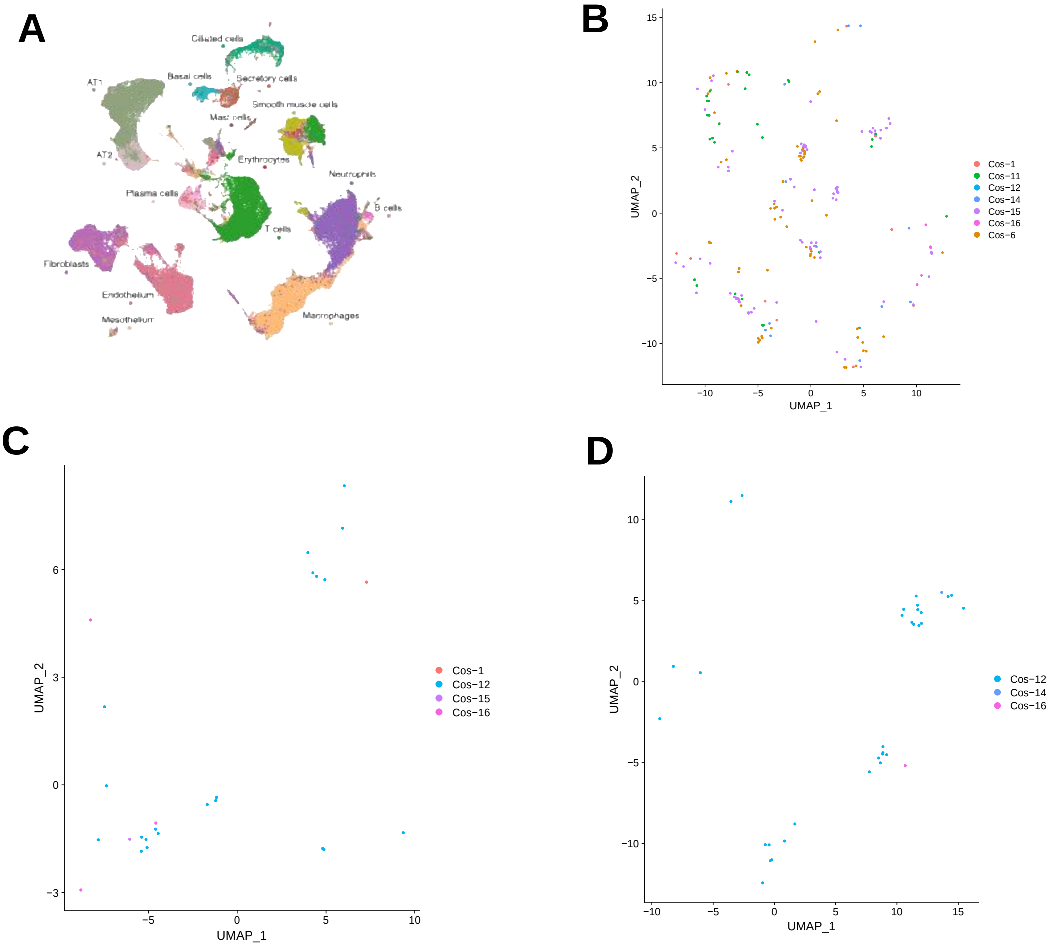

E

Tissue	Lung	Blood	Nasal	Total
Cos1	10	1	X	11
Cos6	69	X	X	69
Cos11	30	X	X	30
Cos12	1	21	32	54
Cos14	12	X	1	13
Cos15	87	1	X	88
Cos16	5	3	1	9
Total	214	26	34	274

Supplemental Figure 5 | Bulk approaches to explore gene signatures

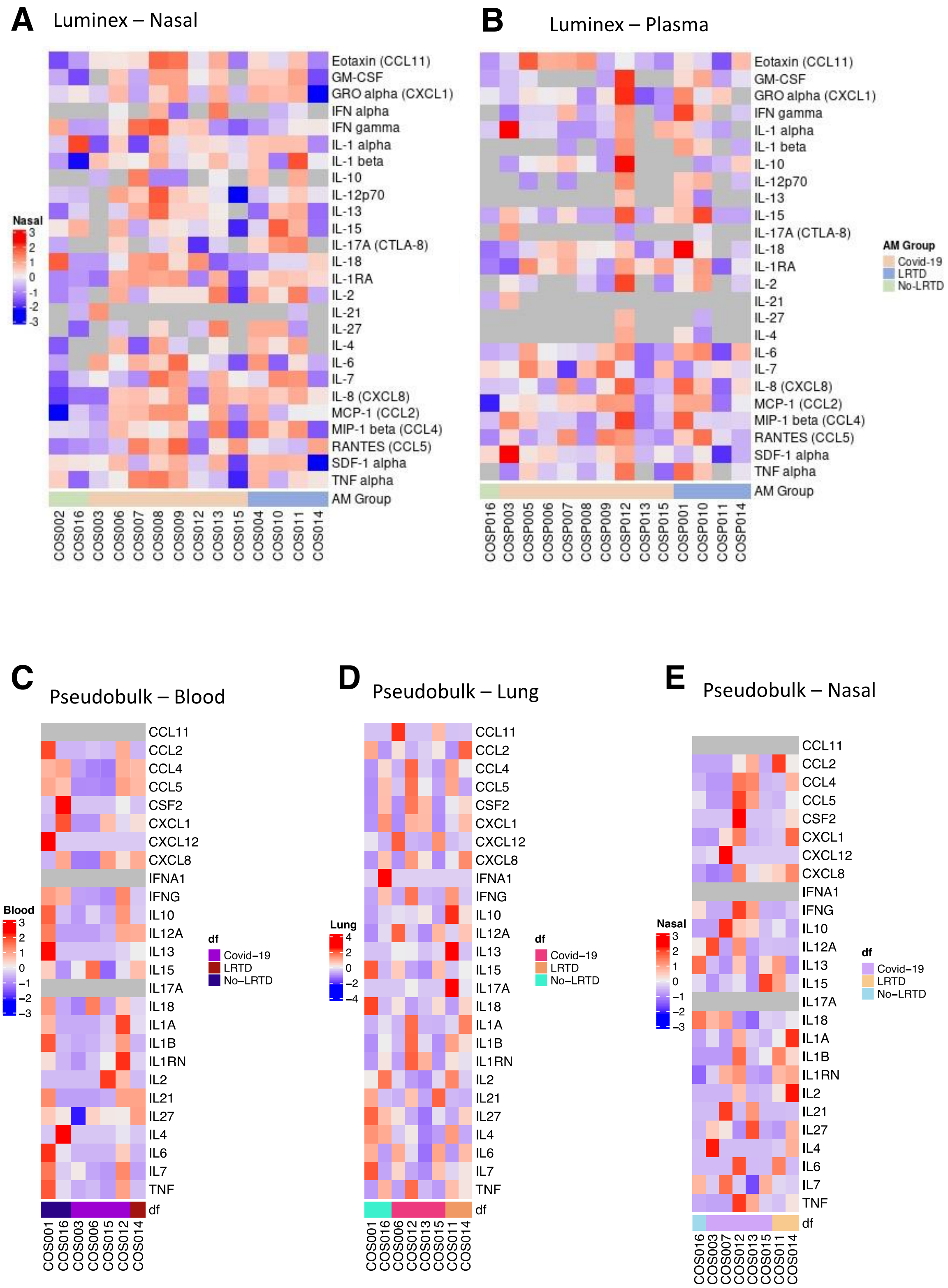

Supplemental Figure 6 | Circos plot showing the top 50 differentially expressed cell:cell interactions upregulated in our COVID-19 cohort compared to LRTD.

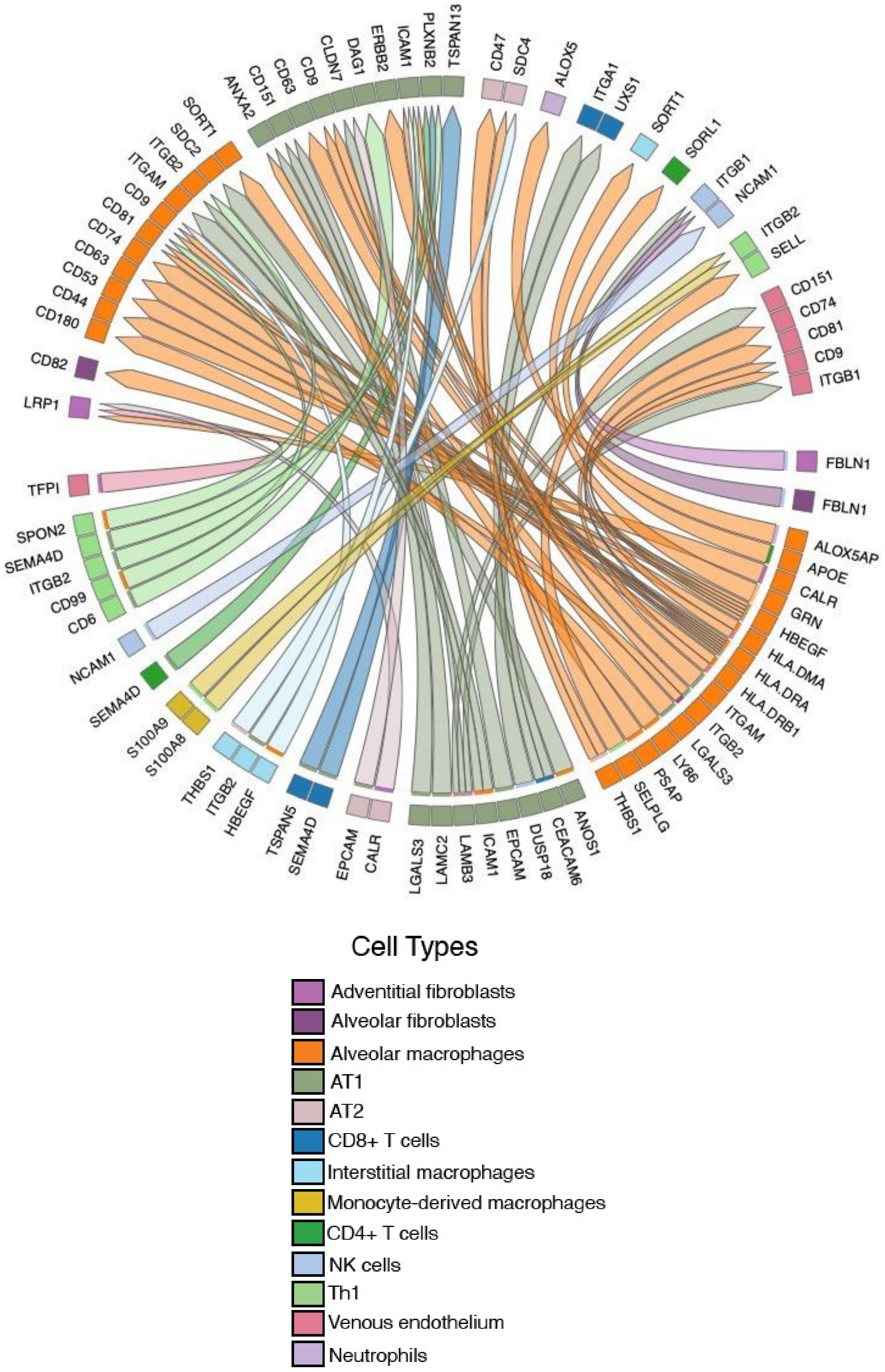

Supplemental Figure 7 | Gene regulatory network of top cellular interactions in Covid-19 compared to LRTD

A

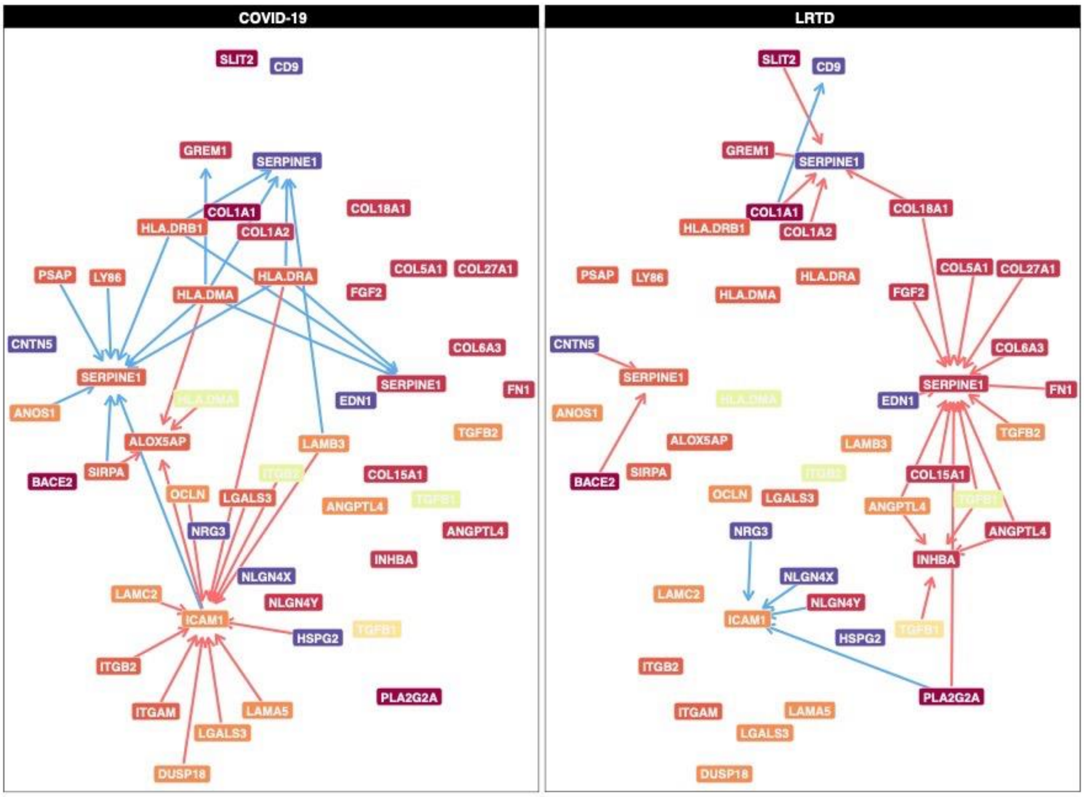

Supplemental Figure 8 | Predicted downstream regulatory targets of cellular crosstalk between lung alveolar macrophages neutrophils and stromal cells in Covid-19 show distinct SPP1 mechanisms

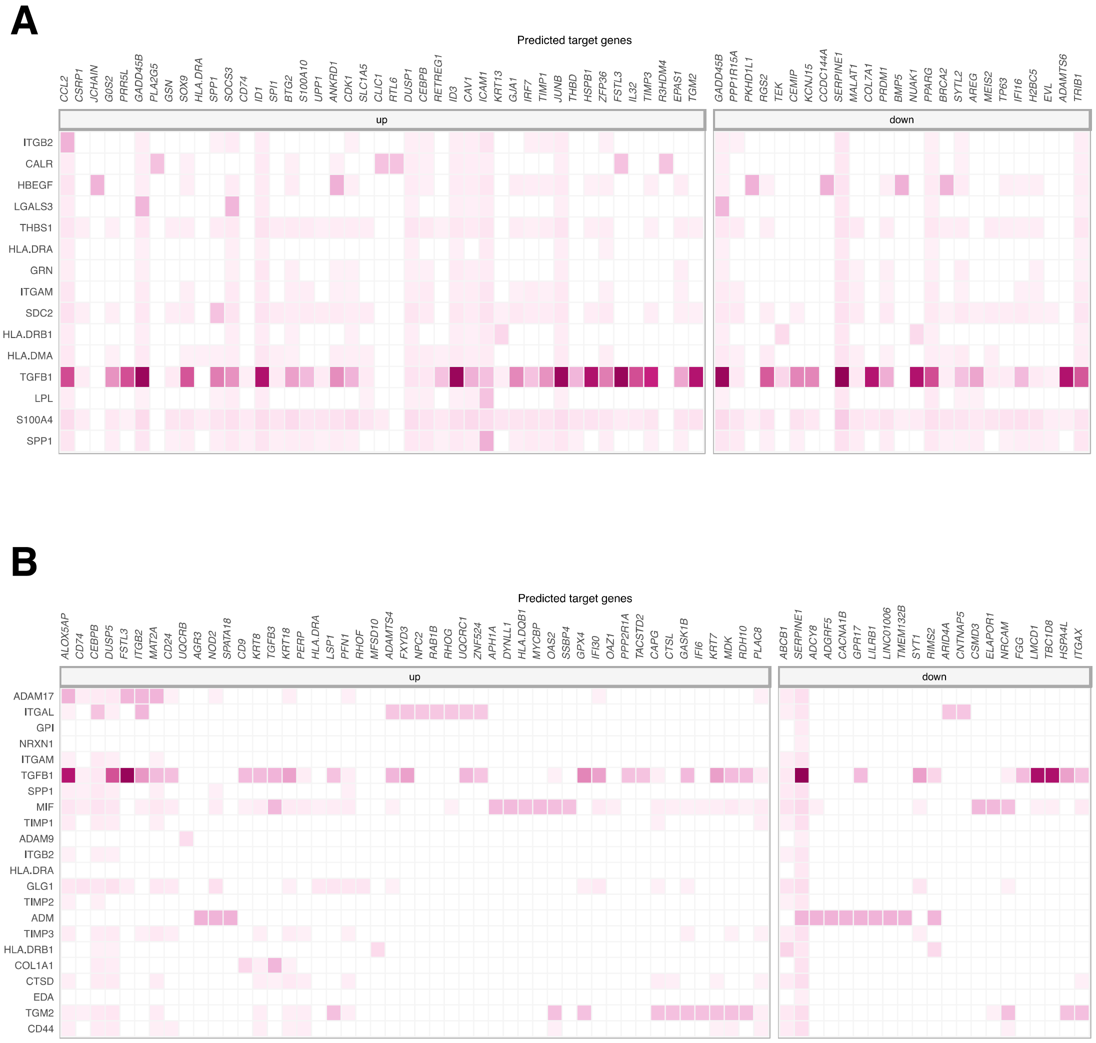

Figure Legends – Supplemental Figures

Supplemental Figure 1 | Histological characteristics of lung pathology in patients with SARS CoV-2 infection. *All images are of Haematoylin and Eosin (H&E) staining of formalin-fixed and paraffin-embedded (FFPE) postmortem lung biopsies from our Malawi cohort.* Images are a selection from reviewed whole slides of left and right lung samples from 16 cases, chosen to highlight specific features of interest. Additional whole slide images are available to view on virtual microscope: https://covid-atlas.cvr.gla.ac.uk **A)** Covid19 cases (Cos08 right lung) shows diffuse alveolar damage with fibrin (*) and accumulation of intra-alveolar macrophages (circles). **B)** Covid19 case (Cos06 left lung) shows severe disruption of the alveoli and the lung architecture with fibrin extravasation (*) and diffuse alveolar damage. The arrow shows a thrombus in a vessel. **C)** Covid19 case (CoS05 left lung), shows proliferation of type 2 pneumocytes (arrows) **D)** Covid19 case (CoS09 right lung) shows thickening of alveolar walls due to inflammatory cells and increased collagen (between the arrows). Proliferation of type 2 pneumocytes is present (*). **E)** Non-Covid19 lower respiratory tract disease cases (LRTD, CoS014 right lung) shows severe infiltration with viable and degenerated neutrophils within the alveolar spaces (*) in most areas admixed with fibrin (**). **F)** Covid19 case (Cos013 right lung) shows lung architecture replaced by irregular proliferation of collagen-rich granulation tissue (*). All bars, 50 micrometres.

Supplemental Figure 2 | Top cluster markers characterising immune and stromal cell populations in the lung in the Malawi cohort.
A) Dotplot showing the average expression of top 3 cluster markers for each cell type in the lung immune compartment (**Fig3B**). **B)** Dotplot showing the average expression of top 3 cluster markers for each cell type in the lung stromal compartment (**Fig3C**).

Supplemental Figure 3 | Characterisation of cell populations in the nasal and peripheral blood tissue compartments in the Malawi cohort.
A) Dotplot showing the average expression of top 5 cluster markers for each cell type in the nasal compartment (**Fig5A**). **B)** Dotplot showing the average expression of top 5 cluster markers for each cell type in the peripheral blood compartment (**Fig5B**). **C-D)** Cell type proportion bar plots of cell types in the nasal and peripheral blood compartment shown in **Fig 5A** and **Fig 5B,** grouped by disease group.

Supplemental Figure 4 | Minimal SARS-CoV2 reads in single-cell data of lung, nasal and blood cells
A) Lung reference as in Fig 3a. **B-D)** UMAPs indicate the cells in which we found reads that mapped to the SARS-CoV2 genome, coloured by case. **E)** Table showing absolute cell numbers per case that contain expression of UMIs that passed quality control steps that map to the SARS-CoV2 genome in the lung, peripheral blood and nasal compartment.

Supplemental Figure 5 | Bulk approaches to explore gene signatures
Heatmaps showing cytokine signatures in different tissues. Values are plotted as z-score (grey mean not measured). Samples are grouped by their disease type. Luminex data of Nasal (A) and Plasma (B). Data were transformed with a log2 and for the visualisation with ComplexHeatmap in R with a Z-score by gene. For the statistical tests we compared levels of IFN-y, IL6, IL8, TNF and IL1b in nasal fluid and plasma between the Covid-19 and LTRD

samples using a Welch Two Sample t-test which was non-significant for all comparisons, we did not correct for multiple comparisons. (C-E) Pseudobulk heatmaps showing cytokines included in the Luminex panel on the transcriptomic level in the peripheral blood, lung and nasal compartment per patient. As for Luminex we compared levels of IFN-y, IL6, IL8, TNF and IL1b in nasal, blood and lung cells between the Covid-19 and LTRD samples using a Welch Two Sample t-test which was non-significant for all comparisons, we did not correct for multiple comparisons.

Supplemental Figure 6 | Circos plot showing the top 50 differentially expressed cell:cell interactions upregulated in our COVID-19 cohort compared to LRTD.
Segments are coloured by cell type with ligands and receptors labelled on the outside. Direction of the arrows show the senders of communications i.e. expression of ligand, and receiver of communications. Inner tracks on sender segments are coloured by the receiving cell type for ease of interpretation.

Supplemental Figure 7 | Gene regulatory network of top cellular interactions in Covid-19 compared to LRTD
A) Gene regulatory network showing the top downstream regulatory targets as a consequence of interacting cell types in the lung in COVID-19 compared to LRTD. Red arrows indicate that the gene is being upregulated and blue arrows indicate the gene is being downregulated.

Supplemental Figure 8 | Predicted downstream regulatory targets of cellular crosstalk between lung alveolar macrophages neutrophils and stromal cells in Covid-19 show distinct SPP1 mechanisms
A) Heatmap showing the regulatory potential between the top highly expressed predicted ligands in alveolar macrophages in the lung with downstream intracellular targets in lung stromal cells. **B)** Heatmap showing the regulatory potential between the top highly expressed predicted ligands in neutrophils in the lung with downstream intracellular targets in lung endothelium.

Supplemental Table 1 | *Additional clinical information*
Key is below the table

Supplemental Table 2 | *Ante and postmortem laboratory results*
Key is in second tab

Supplemental Table 3 | *Histopathology scoring for different organs.*
Each organ is in a different tab: Lung, spleen, brain, bone marrow, liver, heart

Supplemental Table 4 | *Comparison of cell proportions in IMC data*
The table is divided into different sections by tabs of the excel file. The clinical groups Malawi tab shows the proportion of different immune, stromal and vascular cells in the three groups in the Malawi cohort: Covid19, LRTD and non-LRTD and statistical comparison between the groups. The HIV tabs shows comparison between HIV positive and HIV negative Covid19 cases in the Malawi cohort. The cohorts tab shows comparison between the three IMC cohorts after integration: Brazil, USA and Malawi. The cohorts and

progression tab shows these same comparisons in integrated data but the cases in each cohort are divided into subgroups based on whether patients died within the first 14 days after illness onset (early death) or more than 14 days after illness onset (late death) this division is only made for the Brazil and USA cohorts as the Malawi cohort had only one case that died after 14 days. The cohorts and variants tab shows these same comparisons in integrated data but where the cases in the Malawi cohort are divided into subgroups based on the relevant viral variant; USA and Brazil cases are not subdivided because all cases were ancestral variant.

Supplemental Table 5 | *Summary statistics of CellRanger output and processing of scRNA/snRNA-sequencing data*
The table is organised into three tabs detailing the mapping summary statistics for each scRNA/snRNA-sequencing run included in the study, quatification and demultiplexing statistics for the hashtagged runs and the summary statistics for the SNP splitting genotype assignment.

Supplemental Table 6 | *Cell counts for single-cell data for lung, nasal and blood cells from the Malawian cohort split by disease group.*
Each tab shows cell counts for different immune and stromal cell types for Covid19, LRTD and non-LRTD cases.

Supplemental Table 7| *Differential gene expression in single-cell data by cell type*
Differential gene expression analysis results from all cell types in the lung, nasal and blood tissue compartments. The table is organised with each comparison of cell types in the Malawi cohort in Covid-19 compared to LRTD in the three tissues and includes comparisons between lung cells in the Malawi cohort compared to the HLCA cohort. The table contains the average log fold change (avg_log2FC) along with the p-value (p_val) and multiple-test corrected p-values (p_val_adj).

Software References

1. S. Berg, D. Kutra, T. Kroeger, C. N. Straehle, B. X. Kausler, C. Haubold, M. Schiegg, J. Ales, T. Beier, M. Rudy, K. Eren, J. I. Cervantes, B. Xu, F. Beuttenmueller, A. Wolny, C. Zhang, U. Koethe, F. A. Hamprecht, A. Kreshuk, ilastik: interactive machine learning for (bio)image analysis. *Nat Methods* **16**, 1226-1232 (2019); published online EpubDec (10.1038/s41592-019-0582-9).
2. J. J. Sebastien Le, Francois Husson FactoMineR: An R Package for Multivariate Analysis. *Journal of Statistical Software* **25**, 1-18 (2008)10.18637/jss.v025.i01).
3. C. M. Josse J, Liquet B, Husson F, Handling Missing Values with Regularized Iterative Multiple Correspondence Analysis. *Journal of Classification* **29**, 91-116 (2012)10.1007/s00357-012-9097-0).
4. N. D. Nils Eling, Tobias Hoch, Bernd Bodenmiller cytomapper: an R/Bioconductor package for visualisation of highly multiplexed imaging data. *Bioinformatics*, (2020)10.1093/bioinformatics/btaa1061).
5. E. Dann, N. C. Henderson, S. A. Teichmann, M. D. Morgan, J. C. Marioni, Differential abundance testing on single-cell data using k-nearest neighbor graphs. *Nat Biotechnol* **40**, 245-253 (2022); published online EpubFeb (10.1038/s41587-021-01033-z).
6. R. Dries, Q. Zhu, R. Dong, C. L. Eng, H. Li, K. Liu, Y. Fu, T. Zhao, A. Sarkar, F. Bao, R. E. George, N. Pierson, L. Cai, G. C. Yuan, Giotto: a toolbox for integrative analysis and visualization of spatial expression data. *Genome Biol* **22**, 78 (2021); published online EpubMar 8 (10.1186/s13059-021-02286-2).
7. H. W. Jackson, J. r. Fischer, V. r. T. Zanotelli, h. r. ali, r. Mechera, S. D. Soysal, h. Moch, S. Muenst, Z. Varga, W. P. Weber, B. Bodenmiller, The single-cell pathology landscape of breast cancer. Nature 578, 615–620 (2020).
8. P. Lu, K. A. Oetjen, D. E. Bender, M. B. Ruzinova, D. A. C. Fisher, K. G. Shim, R. K. Pachynski, W. N. Brennen, S. T. Oh, D. C. Link, D. L. J. Thorek, IMC-Denoise: a content aware denoising pipeline to enhance Imaging Mass Cytometry. *Nat Commun* **14**, 1601 (2023); published online EpubMar 23 (10.1038/s41467-023-37123-6).
9. N. F. Greenwald, G. Miller, E. Moen, A. Kong, A. Kagel, T. Dougherty, C. C. Fullaway, B. J. McIntosh, K. X. Leow, M. S. Schwartz, C. Pavelchek, S. Cui, I. Camplisson, O. Bar-Tal, J. Singh, M. Fong, G. Chaudhry, Z. Abraham, J. Moseley, S. Warshawsky, E. Soon,

S. Greenbaum, T. Risom, T. Hollmann, S. C. Bendall, L. Keren, W. Graf, M. Angelo, D. Van Valen, Whole-cell segmentation of tissue images with human-level performance using large-scale data annotation and deep learning. *Nat Biotechnol* **40**, 555-565 (2022); published online EpubApr (10.1038/s41587-021-01094-0).

10. F. A. Wolf, P. Angerer, F. J. Theis, SCANPY: large-scale single-cell gene expression data analysis. *Genome Biol* **19**, 15 (2018); published online EpubFeb 6 (10.1186/s13059-017-1382-0).
11. L. A. H. McInnes, John and Saul, Nathaniel and Grossberger, Lukas, UMAP: Uniform Manifold Approximation and Projection. *The Journal of Open Source Software* **3**, 861 (2018).
12. M. J. Geuenich, J. Hou, S. Lee, S. Ayub, H. W. Jackson, K. R. Campbell, Automated assignment of cell identity from single-cell multiplexed imaging and proteomic data. *Cell Syst* **12**, 1173-1186 e1175 (2021); published online EpubDec 15 (10.1016/j.cels.2021.08.012).
13. G. Palla, H. Spitzer, M. Klein, D. Fischer, A. C. Schaar, L. B. Kuemmerle, S. Rybakov, I. L. Ibarra, O. Holmberg, I. Virshup, M. Lotfollahi, S. Richter, F. J. Theis, Squidpy: a scalable framework for spatial omics analysis. *Nat Methods* **19**, 171-178 (2022); published online EpubFeb (10.1038/s41592-021-01358-2).
14. A. L. Martinelli, M. A. Rapsomaniki, ATHENA: analysis of tumor heterogeneity from spatial omics measurements. *Bioinformatics* **38**, 3151-3153 (2022); published online EpubMay 26 (10.1093/bioinformatics/btac303).
15. P. Weeratunga, l. Denney, J. a. Bull, e. repapi, M. Sergeant, r. etherington c. Vuppussetty, G. D. h. Turner, c. clelland, a. cross, F. issa, c. e. de andrea, i. M. Bermejo, D. Sims, S. McGowan, Y.-X. Zurke, D. J. ahern, e. c. Gamez, J. Whalley, D. richards, P. Klenerman, c. Monaco, i. a. Udalova, T. Dong, G. ogg, J. c. Knight, h. M. Byrne, S. Taylor, l.-P. ho, Single cell spatial analysis reveals inflammatory foci of immature neutrophil and cD8 T cells in coViD-19 lungs. Nat. Comm. 14, 7216 (2023).
