# Supplementary material for: Spatially resolved single-cell atlas unveils a distinct cellular signature of fatal lung COVID-19 in a Malawian population
Source: Nat Med. 2024 Nov 20;30(12):3765–77. doi: 10.1038/s41591-024-03354-3 (PMC11645280; doi:10.1038/s41591-024-03354-3)
Supplement: Supplementary file 2 — Reporting Summary [file 41591_2024_3354_MOESM2_ESM.pdf]

nature portfolio

Corresponding author(s): Christopher Moxon, Matthias Marti, Thomas Otto

Last updated by author(s): Oct 6, 2024

# Reporting Summary

Nature Portfolio wishes to improve the reproducibility of the work that we publish. This form provides structure for consistency and transparency in reporting. For further information on Nature Portfolio policies, see our Editorial Policies and the Editorial Policy Checklist.

## Statistics

For all statistical analyses, confirm that the following items are present in the figure legend, table legend, main text, or Methods section.

| n/a | Confirmed | |
|---|---|---|
| ☐ | ☒ | The exact sample size (*n*) for each experimental group/condition, given as a discrete number and unit of measurement |
| ☐ | ☒ | A statement on whether measurements were taken from distinct samples or whether the same sample was measured repeatedly |
| ☐ | ☒ | The statistical test(s) used AND whether they are one- or two-sided<br>*Only common tests should be described solely by name; describe more complex techniques in the Methods section.* |
| ☐ | ☒ | A description of all covariates tested |
| ☐ | ☒ | A description of any assumptions or corrections, such as tests of normality and adjustment for multiple comparisons |
| ☐ | ☒ | A full description of the statistical parameters including central tendency (e.g. means) or other basic estimates (e.g. regression coefficient) AND variation (e.g. standard deviation) or associated estimates of uncertainty (e.g. confidence intervals) |
| ☐ | ☒ | For null hypothesis testing, the test statistic (e.g. *F*, *t*, *r*) with confidence intervals, effect sizes, degrees of freedom and *P* value noted<br>*Give P values as exact values whenever suitable.* |
| ☒ | ☐ | For Bayesian analysis, information on the choice of priors and Markov chain Monte Carlo settings |
| ☒ | ☐ | For hierarchical and complex designs, identification of the appropriate level for tests and full reporting of outcomes |
| ☐ | ☒ | Estimates of effect sizes (e.g. Cohen's *d*, Pearson's *r*), indicating how they were calculated |

*Our web collection on statistics for biologists contains articles on many of the points above.*

## Software and code

Policy information about availability of computer code

| | |
|---|---|
| Data collection | Data were collected using clinical reporting forms and entered into REDCap |
| Data analysis | umap-learn Python package, v 0.5.3; ASsignmenT of sIngle-cell pRoteomics v 0.1.4; miloR R package (v 1.4.0); Spatial Quantification of Molecular Data in Python v. 1.2.2;Seurat; multinichenetR; MaxFuse v0.0.2<br><br>All code and software used in the analysis are detailed in our GitHub repositories: https://github.com/olympiahardy/COSMIC_Malawi_Covid_Atlas and https://github.com/joaolsf/Spatial_Single_Cell_Lung_Atlas_Malawi_COVID |

For manuscripts utilizing custom algorithms or software that are central to the research but not yet described in published literature, software must be made available to editors and reviewers. We strongly encourage code deposition in a community repository (e.g. GitHub). See the Nature Portfolio guidelines for submitting code & software for further information.

# Data

Policy information about availability of data

All manuscripts must include a data availability statement. This statement should provide the following information, where applicable:

- Accession codes, unique identifiers, or web links for publicly available datasets
- A description of any restrictions on data availability
- For clinical datasets or third party data, please ensure that the statement adheres to our policy

scRNA-Seq: Raw data and processed count matrices are deposited at the EBI ArrayExpress (Accession number E-MTAB-13544 (private until publication). Fully processed objects are deposited on Zenodo 10.5281/zenodo.13898422 and here 10.5281/zenodo.13899297 for the scRNA-seq and IMC respectively.
Lung Atlas - https://cellatlas-cxg.mvls.gla.ac.uk/COSMIC/view/COSMIC_Lung_Atlas.h5ad/
Lung Immune Atlas - https://cellatlas-cxg.mvls.gla.ac.uk/COSMIC/view/COSMIC_Lung_Immune_Atlas.h5ad/
Lung Stromal Atlas - https://cellatlas-cxg.mvls.gla.ac.uk/COSMIC/view/COSMIC_Lung_Stromal_Atlas.h5ad/
Nasal Atlas - https://cellatlas-cxg.mvls.gla.ac.uk/COSMIC/view/COSMIC_Nasal_Atlas.h5ad/
Blood Atlas - https://cellatlas-cxg.mvls.gla.ac.uk/COSMIC/view/COSMIC_Blood_Atlas.h5ad/
Histopathology slides on virtual microscope: https://covid-atlas.cvr.gla.ac.uk
Metadata for the cases (without identifying information) is provided in Extended Table 1.
IMC lung atlas - https://cellatlas-cxg.mvls.gla.ac.uk/COSMIC/view/COSMIC_IMC_Lung.h5ad/

# Research involving human participants, their data, or biological material

Policy information about studies with human participants or human data. See also policy information about sex, gender (identity/presentation), and sexual orientation and race, ethnicity and racism.

| | |
|---|---|
| Reporting on sex and gender | A priori we aimed to recruit fatal cases with an even sex ratio. The sex of cases and proportions of cases in each group that were male and female sex are indicated in the summary table and the sex of cases is indicated in metadata linked to single cell and imaging mass cytometry data. |
| Reporting on race, ethnicity, or other socially relevant groupings | All the cases in our study were of African ethnic background and residents in the Southern region of Malawi |
| Population characteristics | These are summarized in Extended data table 1 and supplementary table 1 |
| Recruitment | We recruited patients aged 45-75 admitted to Queen Elizabeth Central Hospital, Blantyre between October 2020 and July 2021 during which there were two epidemiological waves driven by different SARS-CoV2 variants: Beta (Dec 2020-Feb 2021) and Delta (May-July 2021). Patients admitted with respiratory signs were routinely tested for SARS-CoV2 at QECH. We recruited cases into three groups based on clinical criteria: 1) a Covid19 group (n=9) with clinical features suggesting acute respiratory distress (ARDS, oxygen requirement and either respiratory signs on clinical examination or chest x-ray changes or both) and who had at least one nasal swab positive for SARS-CoV2 on admission; 2) A non-Covid19 LRTD (lower respiratory tract disease) group (n=5) who had clinical signs of ARDS but were negative for SARS-CoV-2 on admission and during hospitalisation; 3) a no LRTD, COVID-19 negative group (n=2) who had no oxygen requirement and no clinical signs of LRTD and for whom the admission and any subsequent nasal swabs were negative for SARS-CoV2 on PCR (Fig.1b, Extended Data Table 1). The study only recruited cases who died between 12 midnight and 12 noon to minimize the postmortem interval and to avoid doing any autopsies at night. We also aimed to balance sex and HIV status so had to decline some eligible male and some HIV positive cases. Otherwise our cases broadly reflect the demographics of the severe cases on our ward during this period. We do not think this selection introduced systematic bias, but cannot exclude this possibility in this small cohort. |
| Ethics oversight | The study protocol was approved by the National Health Scientific Research Committee (NHSRC) in Malawi (Protocol number 07/09/1913) and and by the Medical Veterinary Life Sciences ethics committee in Glasgow (protocol number 200190041) |

Note that full information on the approval of the study protocol must also be provided in the manuscript.

# Field-specific reporting

Please select the one below that is the best fit for your research. If you are not sure, read the appropriate sections before making your selection.

☒ Life sciences ☐ Behavioural & social sciences ☐ Ecological, evolutionary & environmental sciences

For a reference copy of the document with all sections, see nature.com/documents/nr-reporting-summary-flat.pdf

# Life sciences study design

All studies must disclose on these points even when the disclosure is negative.

| | |
|---|---|
| Sample size | No statistical method was used to predetermine sample size. 16 cases in total were recruited across the three clinical groups with this number determined by logistical feasibility and by funding levels. These numbers were considered to be sufficient for these planned single cells and high-dimensional analysis based on sample sizes in other published studies using similar methods. |

Data exclusions

We excluded 9 single cell sequencing runs that had few to no cells and that did not pass standard QC metrics. Within our lung atlas a population of cells (n=1348) were excluded that we deemed to be low quality cells that almost exclusively derived from one multiplexed single nuclei sequencing run that exhibited extremely low UMI counts. 2 non-Covid19 LRTD cases were excluded from IMC runs as they had evidence of active TB lung disease because of theoretical safety concerns as IMC can generate aerosol.

Replication

Not applicable as data were based on human samples, however key findings were validated using orthogonal methods.

Randomization

The experiments were not randomized.

Blinding

Scoring by pathologists and validation experiments were done with investigators blinded to patient group.

# Reporting for specific materials, systems and methods

We require information from authors about some types of materials, experimental systems and methods used in many studies. Here, indicate whether each material, system or method listed is relevant to your study. If you are not sure if a list item applies to your research, read the appropriate section before selecting a response.

## Materials & experimental systems

| n/a | Involved in the study |
|---|---|
| ☐ | ☒ Antibodies |
| ☐ | ☐ Eukaryotic cell lines |
| ☐ | ☐ Palaeontology and archaeology |
| ☐ | ☐ Animals and other organisms |
| ☐ | ☒ Clinical data |
| ☐ | ☐ Dual use research of concern |
| ☐ | ☐ Plants |

## Methods

| n/a | Involved in the study |
|---|---|
| ☐ | ☐ ChIP-seq |
| ☐ | ☐ Flow cytometry |
| ☐ | ☐ MRI-based neuroimaging |

## Antibodies

Antibodies used

Smooth muscle actin (Bio-Rad, Clone: 1A4, Catalogue#: MCA5781GA, Stock conc: 0.66 mg/ml, Dilution: 50) Metal channel: 89
Cd68 (Thermo, Clone: KP1, Catalogue#: MA5-13324, Stock conc: 0.48 mg/ml, Dilution: 50) Metal channel: 113
Cd235ab (BioLegend, Clone: HIR2, Catalogue#: 306615, Stock conc: 0.5 mg/ml, Dilution: 200) Metal channel: 115
Pan-cytokeratin (Biolegend, Clone: AE-1/AE-3, Catalogue#: 914204, Stock conc: 0.63 mg/ml, Dilution: 50) Metal channel: 139
Cd38 (Standard Biotools, Clone: EPR4106, Catalogue#: 3141018D, Stock conc: 0.5 mg/ml, Dilution: 50) Metal channel: 141
MHC-1 (Abcam, Clone: EMR8-5, Catalogue#: ab70328, Stock conc: 0.27 mg/ml, Dilution: 50) Metal channel: 142
Vimentin (Standard Biotools, Clone: RV202, Catalogue#: 3143029D, Stock conc: 0.5 mg/ml, Dilution: 100) Metal channel: 143
Cd14 (Cell Signalling, Clone: D7A2T, Catalogue#: 56082BF, Stock conc: 0.65 mg/ml, Dilution: 50) Metal channel: 144
ICAM1 (Abcam, Clone: EP1442Y, Catalogue#: ab271852, Stock conc: 0.47 mg/ml, Dilution: 50) Metal channel: 145
Cd16 (Abcam, Clone: SP175, Catalogue#: ab243925-100ug, Stock conc: 0.5 mg/ml, Dilution: 50) Metal channel: 146
iNOS (Abcam, Clone: SP126, Catalogue#: ab239990, Stock conc: 0.4 mg/ml, Dilution: 50) Metal channel: 147
Cd66b (Novus, Clone: G10F5, Catalogue#: g10f5_nb100-77808, Stock conc: 0.41 mg/ml, Dilution: 50) Metal channel: 148
Cd11b (Abcam, Clone: EP1345Y, Catalogue#: ab52478, Stock conc: 0.5 mg/ml, Dilution: 50) Metal channel: 149
Cd44 (BioLegend, Clone: IM7, Catalogue#: 103001, Stock conc: 0.49 mg/ml, Dilution: 100) Metal channel: 150
Cd107a (Standard Biotools, Clone: H4A3, Catalogue#: 3151021D, Stock conc: 0.5 mg/ml, Dilution: 100) Metal channel: 151
Cd45 (eBioscience, Clone: CD45-2B11, Catalogue#: 14-9457-82, Stock conc: 0.65 mg/ml, Dilution: 50) Metal channel: 152
Cd31 (Novus, Clone: JC/70A, Catalogue#: jc-70a_nb600-562, Stock conc: 0.53 mg/ml, Dilution: 50) Metal channel: 153
Cd11c (Standard Biotools, Clone: Polyclonal, Catalogue#: 3154025D, Stock conc: 0.5 mg/ml, Dilution: 100) Metal channel: 154
Foxp3 (Abcam, Clone: 236A/E7, Catalogue#: ab20034, Stock conc: 0.5 mg/ml, Dilution: 50) Metal channel: 155
Cd4 (Standard Biotools, Clone: EPR6855, Catalogue#: 3156033D, Stock conc: 0.5 mg/ml, Dilution: 50) Metal channel: 156
SARS-Cov2 (Novus, Clone: Polyclonal, Catalogue#: NB100-56576, Stock conc: 0.5 mg/ml, Dilution: 25) Metal channel: 158
Von Willebrand Factor (Dako, Clone: Polyclonal, Catalogue#: A0082, Stock conc: 1.27 mg/ml, Dilution: 100) Metal channel: 159
Vista (Standard Biotools, Clone: D1L2G, Catalogue#: 3160025D, Stock conc: 0.5 mg/ml, Dilution: 50) Metal channel: 160
Cd20 (Standard Biotools, Clone: H1, Catalogue#: 3161029D, Stock conc: 0.5 mg/ml, Dilution: 50) Metal channel: 161
Cd8 (eBioscience, Clone: CD8/144B, Catalogue#: 14-0085-82, Stock conc: 0.55 mg/ml, Dilution: 50) Metal channel: 162
iba1 (WAKO, Clone: Polyclonal, Catalogue#: 019-19741, Stock conc: 0.42 mg/ml, Dilution: 100) Metal channel: 163
Arginase-1 (Standard Biotools, Clone: D4E3M, Catalogue#: 3164027D, Stock conc: 0.5 mg/ml, Dilution: 50) Metal channel: 164
Fibrinogen (Abcam, Clone: EPR18145-84, Catalogue#: ab227063, Stock conc: 2.93 mg/ml, Dilution: 100) Metal channel: 165
Cd74 (Standard Biotools, Clone: LN2, Catalogue#: 3166025D, Stock conc: 0.5 mg/ml, Dilution: 50) Metal channel: 166
Granzyme B (Standard Biotools, Clone: EPR20129-217, Catalogue#: 3167021D, Stock conc: 0.5 mg/ml, Dilution: 100) Metal channel: 167
Ki-67 (Abcam, Clone: B56, Catalogue#: ab279657, Stock conc: 0.56 mg/ml, Dilution: 100) Metal channel: 168
Collagen Type I (Standard Biotools, Clone: Polyclonal, Catalogue#: 3169023D, Stock conc: 0.5 mg/ml, Dilution: 100) Metal channel: 169
Cd3 (Cell Signalling, Clone: D7A6E, Catalogue#: 85061BF, Stock conc: 0.53 mg/ml, Dilution: 50) Metal channel: 170
pERK1/2 [T202/Y204] (Standard Biotools, Clone: D13.14.4E, Catalogue#: 3171021D, Stock conc: 0.5 mg/ml, Dilution: 50) Metal channel: 171
Cleaved Caspase 3 (Standard Biotools, Clone: 5A1E, Catalogue#: 3172023A, Stock conc: 0.5 mg/ml, Dilution: 50) Metal channel: 172
CD45RO (Standard Biotools, Clone: UCHL1, Catalogue#: 3173016D, Stock conc: 0.5 mg/ml, Dilution: 50) Metal channel: 173

MHC2 (Abcam, Clone: TAL1B5, Catalogue#: ab176408, Stock conc: 0.5 mg/ml, Dilution: 50) Metal channel: 174
Cd206 (Cell Signalling, Clone: E2L9N, Catalogue#: 91992, Stock conc: 0.43 mg/ml, Dilution: 50) Metal channel: 175
Cd163 (Bio-Rad, Clone: EDHu-1, Catalogue#: MCA1853, Stock conc: 0.9 mg/ml, Dilution: 50) Metal channel: 196

Validation

Antibodies for immune markers were optimized and validated on formalin-fixed paraffin embedded human lymph nodes. SARS-Cov2 staining was previously validated in COVID lungs from Brazilian cohort, using lungs from pneumonia patients and control lungs as negative controls.

## Eukaryotic cell lines

Policy information about cell lines and Sex and Gender in Research

Cell line source(s)

*State the source of each cell line used and the sex of all primary cell lines and cells derived from human participants or vertebrate models.*

Authentication

*Describe the authentication procedures for each cell line used OR declare that none of the cell lines used were authenticated.*

Mycoplasma contamination

*Confirm that all cell lines tested negative for mycoplasma contamination OR describe the results of the testing for mycoplasma contamination OR declare that the cell lines were not tested for mycoplasma contamination.*

Commonly misidentified lines
(See ICLAC register)

*Name any commonly misidentified cell lines used in the study and provide a rationale for their use.*

## Palaeontology and Archaeology

Specimen provenance

*Provide provenance information for specimens and describe permits that were obtained for the work (including the name of the issuing authority, the date of issue, and any identifying information). Permits should encompass collection and, where applicable, export.*

Specimen deposition

*Indicate where the specimens have been deposited to permit free access by other researchers.*

Dating methods

*If new dates are provided, describe how they were obtained (e.g. collection, storage, sample pretreatment and measurement), where they were obtained (i.e. lab name), the calibration program and the protocol for quality assurance OR state that no new dates are provided.*

☐ Tick this box to confirm that the raw and calibrated dates are available in the paper or in Supplementary Information.

Ethics oversight

*Identify the organization(s) that approved or provided guidance on the study protocol, OR state that no ethical approval or guidance was required and explain why not.*

Note that full information on the approval of the study protocol must also be provided in the manuscript.

## Animals and other research organisms

Policy information about studies involving animals; ARRIVE guidelines recommended for reporting animal research, and Sex and Gender in Research

Laboratory animals

*For laboratory animals, report species, strain and age OR state that the study did not involve laboratory animals.*

Wild animals

*Provide details on animals observed in or captured in the field; report species and age where possible. Describe how animals were caught and transported and what happened to captive animals after the study (if killed, explain why and describe method; if released, say where and when) OR state that the study did not involve wild animals.*

Reporting on sex

*Indicate if findings apply to only one sex; describe whether sex was considered in study design, methods used for assigning sex. Provide data disaggregated for sex where this information has been collected in the source data as appropriate; provide overall numbers in this Reporting Summary. Please state if this information has not been collected. Report sex-based analyses where performed, justify reasons for lack of sex-based analysis.*

Field-collected samples

*For laboratory work with field-collected samples, describe all relevant parameters such as housing, maintenance, temperature, photoperiod and end-of-experiment protocol OR state that the study did not involve samples collected from the field.*

Ethics oversight

*Identify the organization(s) that approved or provided guidance on the study protocol, OR state that no ethical approval or guidance was required and explain why not.*

Note that full information on the approval of the study protocol must also be provided in the manuscript.

# Clinical data

Policy information about clinical studies

All manuscripts should comply with the ICMJE guidelines for publication of clinical research and a completed CONSORT checklist must be included with all submissions.

Clinical trial registration: *Provide the trial registration number from ClinicalTrials.gov or an equivalent agency.*

Study protocol: *Note where the full trial protocol can be accessed OR if not available, explain why.*

Data collection: *Describe the settings and locales of data collection, noting the time periods of recruitment and data collection.*

Outcomes: *Describe how you pre-defined primary and secondary outcome measures and how you assessed these measures.*

# Dual use research of concern

Policy information about dual use research of concern

## Hazards

Could the accidental, deliberate or reckless misuse of agents or technologies generated in the work, or the application of information presented in the manuscript, pose a threat to:

| No | Yes | |
|---|---|---|
| ☒ | ☐ | Public health |
| ☒ | ☐ | National security |
| ☒ | ☐ | Crops and/or livestock |
| ☒ | ☐ | Ecosystems |
| ☒ | ☐ | Any other significant area |

## Experiments of concern

Does the work involve any of these experiments of concern:

| No | Yes | |
|---|---|---|
| ☒ | ☐ | Demonstrate how to render a vaccine ineffective |
| ☒ | ☐ | Confer resistance to therapeutically useful antibiotics or antiviral agents |
| ☒ | ☐ | Enhance the virulence of a pathogen or render a nonpathogen virulent |
| ☒ | ☐ | Increase transmissibility of a pathogen |
| ☒ | ☐ | Alter the host range of a pathogen |
| ☒ | ☐ | Enable evasion of diagnostic/detection modalities |
| ☒ | ☐ | Enable the weaponization of a biological agent or toxin |
| ☒ | ☐ | Any other potentially harmful combination of experiments and agents |

# Plants

Seed stocks: *Report on the source of all seed stocks or other plant material used. If applicable, state the seed stock centre and catalogue number. If plant specimens were collected from the field, describe the collection location, date and sampling procedures.*

Novel plant genotypes: *Describe the methods by which all novel plant genotypes were produced. This includes those generated by transgenic approaches, gene editing, chemical/radiation-based mutagenesis and hybridization. For transgenic lines, describe the transformation method, the number of independent lines analyzed and the generation upon which experiments were performed. For gene-edited lines, describe the editor used, the endogenous sequence targeted for editing, the targeting guide RNA sequence (if applicable) and how the editor was applied.*

Authentication: *Describe any authentication procedures for each seed stock used or novel genotype generated. Describe any experiments used to assess the effect of a mutation and, where applicable, how potential secondary effects (e.g. second site T-DNA insertions, mosiacism, off-target gene editing) were examined.*

## ChIP-seq

### Data deposition

☐ Confirm that both raw and final processed data have been deposited in a public database such as GEO.

☐ Confirm that you have deposited or provided access to graph files (e.g. BED files) for the called peaks.

| | |
|---|---|
| Data access links<br>*May remain private before publication.* | *For "Initial submission" or "Revised version" documents, provide reviewer access links. For your "Final submission" document, provide a link to the deposited data.* |
| Files in database submission | *Provide a list of all files available in the database submission.* |
| Genome browser session<br>(e.g. UCSC) | *Provide a link to an anonymized genome browser session for "Initial submission" and "Revised version" documents only, to enable peer review. Write "no longer applicable" for "Final submission" documents.* |

### Methodology

| | |
|---|---|
| Replicates | *Describe the experimental replicates, specifying number, type and replicate agreement.* |
| Sequencing depth | *Describe the sequencing depth for each experiment, providing the total number of reads, uniquely mapped reads, length of reads and whether they were paired- or single-end.* |
| Antibodies | *Describe the antibodies used for the ChIP-seq experiments; as applicable, provide supplier name, catalog number, clone name, and lot number.* |
| Peak calling parameters | *Specify the command line program and parameters used for read mapping and peak calling, including the ChIP, control and index files used.* |
| Data quality | *Describe the methods used to ensure data quality in full detail, including how many peaks are at FDR 5% and above 5-fold enrichment.* |
| Software | *Describe the software used to collect and analyze the ChIP-seq data. For custom code that has been deposited into a community repository, provide accession details.* |

## Flow Cytometry

### Plots

Confirm that:

☐ The axis labels state the marker and fluorochrome used (e.g. CD4-FITC).

☐ The axis scales are clearly visible. Include numbers along axes only for bottom left plot of group (a 'group' is an analysis of identical markers).

☐ All plots are contour plots with outliers or pseudocolor plots.

☐ A numerical value for number of cells or percentage (with statistics) is provided.

### Methodology

| | |
|---|---|
| Sample preparation | *Describe the sample preparation, detailing the biological source of the cells and any tissue processing steps used.* |
| Instrument | *Identify the instrument used for data collection, specifying make and model number.* |
| Software | *Describe the software used to collect and analyze the flow cytometry data. For custom code that has been deposited into a community repository, provide accession details.* |
| Cell population abundance | *Describe the abundance of the relevant cell populations within post-sort fractions, providing details on the purity of the samples and how it was determined.* |
| Gating strategy | *Describe the gating strategy used for all relevant experiments, specifying the preliminary FSC/SSC gates of the starting cell population, indicating where boundaries between "positive" and "negative" staining cell populations are defined.* |

☐ Tick this box to confirm that a figure exemplifying the gating strategy is provided in the Supplementary Information.

## Magnetic resonance imaging

### Experimental design

| | |
|---|---|
| Design type | *Indicate task or resting state; event-related or block design.* |

Design specifications | *Specify the number of blocks, trials or experimental units per session and/or subject, and specify the length of each trial or block (if trials are blocked) and interval between trials.*

Behavioral performance measures | *State number and/or type of variables recorded (e.g. correct button press, response time) and what statistics were used to establish that the subjects were performing the task as expected (e.g. mean, range, and/or standard deviation across subjects).*

## Acquisition

Imaging type(s) | *Specify: functional, structural, diffusion, perfusion.*

Field strength | *Specify in Tesla*

Sequence & imaging parameters | *Specify the pulse sequence type (gradient echo, spin echo, etc.), imaging type (EPI, spiral, etc.), field of view, matrix size, slice thickness, orientation and TE/TR/flip angle.*

Area of acquisition | *State whether a whole brain scan was used OR define the area of acquisition, describing how the region was determined.*

Diffusion MRI ☐ Used ☐ Not used

## Preprocessing

Preprocessing software | *Provide detail on software version and revision number and on specific parameters (model/functions, brain extraction, segmentation, smoothing kernel size, etc.).*

Normalization | *If data were normalized/standardized, describe the approach(es): specify linear or non-linear and define image types used for transformation OR indicate that data were not normalized and explain rationale for lack of normalization.*

Normalization template | *Describe the template used for normalization/transformation, specifying subject space or group standardized space (e.g. original Talairach, MNI305, ICBM152) OR indicate that the data were not normalized.*

Noise and artifact removal | *Describe your procedure(s) for artifact and structured noise removal, specifying motion parameters, tissue signals and physiological signals (heart rate, respiration).*

Volume censoring | *Define your software and/or method and criteria for volume censoring, and state the extent of such censoring.*

## Statistical modeling & inference

Model type and settings | *Specify type (mass univariate, multivariate, RSA, predictive, etc.) and describe essential details of the model at the first and second levels (e.g. fixed, random or mixed effects; drift or auto-correlation).*

Effect(s) tested | *Define precise effect in terms of the task or stimulus conditions instead of psychological concepts and indicate whether ANOVA or factorial designs were used.*

Specify type of analysis: ☐ Whole brain ☐ ROI-based ☐ Both

Statistic type for inference | *Specify voxel-wise or cluster-wise and report all relevant parameters for cluster-wise methods.*

(See Eklund et al. 2016)

Correction | *Describe the type of correction and how it is obtained for multiple comparisons (e.g. FWE, FDR, permutation or Monte Carlo).*

## Models & analysis

| n/a | Involved in the study |
|---|---|
| ☒ | ☐ Functional and/or effective connectivity |
| ☒ | ☐ Graph analysis |
| ☒ | ☐ Multivariate modeling or predictive analysis |
